# Lipid Composition of the Human Eye: Are Red Blood Cells a Good Mirror of Retinal and Optic Nerve Fatty Acids?

**DOI:** 10.1371/journal.pone.0035102

**Published:** 2012-04-09

**Authors:** Niyazi Acar, Olivier Berdeaux, Stéphane Grégoire, Stéphanie Cabaret, Lucy Martine, Philippe Gain, Gilles Thuret, Catherine P. Creuzot-Garcher, Alain M. Bron, Lionel Bretillon

**Affiliations:** 1 CNRS, UMR6265 Centre des Sciences du Goût et de l'Alimentation, Dijon, France; 2 INRA, UMR1324 Centre des Sciences du Goût et de l'Alimentation, Dijon, France; 3 Université de Bourgogne, UMR Centre des Sciences du Goût et de l'Alimentation, Dijon, France; 4 Biology, Imaging, and Engineering of Corneal Grafts, Faculty of Medicine, Department of Ophthalmology, Saint Etienne, France; 5 Department of Ophthalmology, University Hospital, Dijon, France; University of Tennessee, United States of America

## Abstract

**Background:**

The assessment of blood lipids is very frequent in clinical research as it is assumed to reflect the lipid composition of peripheral tissues. Even well accepted such relationships have never been clearly established. This is particularly true in ophthalmology where the use of blood lipids has become very common following recent data linking lipid intake to ocular health and disease. In the present study, we wanted to determine in humans whether a lipidomic approach based on red blood cells could reveal associations between circulating and tissue lipid profiles. To check if the analytical sensitivity may be of importance in such analyses, we have used a double approach for lipidomics.

**Methodology and Principal Findings:**

Red blood cells, retinas and optic nerves were collected from 9 human donors. The lipidomic analyses on tissues consisted in gas chromatography and liquid chromatography coupled to an electrospray ionization source-mass spectrometer (LC-ESI-MS). Gas chromatography did not reveal any relevant association between circulating and ocular fatty acids except for arachidonic acid whose circulating amounts were positively associated with its levels in the retina and in the optic nerve. In contrast, several significant associations emerged from LC-ESI-MS analyses. Particularly, lipid entities in red blood cells were positively or negatively associated with representative pools of retinal docosahexaenoic acid (DHA), retinal very-long chain polyunsaturated fatty acids (VLC-PUFA) or optic nerve plasmalogens.

**Conclusions and Significance:**

LC-ESI-MS is more appropriate than gas chromatography for lipidomics on red blood cells, and further extrapolation to ocular lipids. The several individual lipid species we have identified are good candidates to represent circulating biomarkers of ocular lipids. However, further investigation is needed before considering them as indexes of disease risk and before using them in clinical studies on optic nerve neuropathies or retinal diseases displaying photoreceptors degeneration.

## Introduction

The nervous system is the organ with the second largest concentration of lipids, only exceeded by adipose tissue. Nervous tissues contain about 50 to 60% of their dry weight as lipids, and approximately 35 to 40% of these lipids are polyunsaturated fatty acids (PUFAs) [1], [2], [3], [4]. These lipids almost exclusively consist in PUFA-rich-membrane phospholipids and in cholesterol playing a structural function without being related to energy metabolism [3].

As elements of the nervous system, ocular tissues such as the optic nerve and the neural retina display similar characteristics [5], [6], [7]. Phospholipids represent about two-thirds of total lipids in these structures and are characterized by species rich in PUFAs. Although docosahexaenoic acid (DHA; C22:6n-3) represents a small percentage of the fatty acids in most human tissues, it is the most abundant PUFA in the retina [7]. The highest level of DHA in the retina is observed in the outer part of photoreceptor cells where it plays important biophysical and biochemical functions in visual transduction [8], and protection against cell injury [9]. Since it is composed by myelinated axons of retinal ganglion cells, the optic nerve is characterized by its high content in particular phospholipids termed as “plasmalogens”, whose concentration is increased in myelin [3], [5], [10].

Ophthalmic diseases affecting the optic nerve and the retina result in alteration of specific cell types, and obviously of the tissue lipid molecular profile. The loss of DHA-rich photoreceptor cells in the retina is a characteristic feature of retinal degenerations such as age-related macular degeneration (AMD) ―the leading cause of vision loss in people aged 65 years or more in western countries [11]― or retinitis pigmentosa that is a group of hereditary retinal degenerations characterized by progressive night blindness [12]. Concerning the optic nerve, glaucomatous optic neuropathy or glaucoma is one of the most important degenerative disease in term of prevalence since it represents the second leading cause of blindness worldwide by affecting more than 60 million people in 2010 [13]. Glaucoma is characterized by structural changes in the optic nerve head and a reduction of the optic nerve diameter, due to a loss of either axons, myelin, or both [14], [15].

Results from numerous epidemiological studies have suggested that lower dietary intakes and lower circulating concentrations of DHA are associated with higher risk of AMD [16], [17], [18], [19], [20], [21]. Several works have also shown lower levels of DHA in plasma and erythrocytes of patients with retinitis pigmentosa [22], [23], [24], [25]. These data led to dietary supplementation trials with DHA for both AMD and retinitis pigmentosa [26], [27], [28], and to the commercialization of DHA-rich dietary supplements in markets [29], the objective being to maintain or to increase retinal DHA in order to prevent these diseases. To our knowledge, only two studies have established potential relationships between circulating lipids and the pathogenesis of glaucoma. Both of them have demonstrated reduced levels of DHA in plasma and red blood cells of primary open angle glaucoma patients [30], [31], together with an additional alteration of circulating levels of plasmalogens [31].

All of these human studies that aim to characterize either pathophysiological events occurring in the eye or the bioavailability/efficacy of dietary treatments need valuable circulating biomarkers in order to mirror retinal concentrations in lipids under pathological influence or in response to a dietary supplementation. Red blood cell membrane lipids are considered as an index of tissue lipid status because the fatty acids they contain are associated with structural membrane lipids [32]. Since red blood cell lipids are also less sensitive than plasma lipids to dietary fluctuations, another advantage is that they represent the longer-term fatty acid status of the individual. A few number of studies have found out some correlations between red blood cell DHA level with DHA concentrations in neural tissues including the retina [4], [33], [34]. However, these studies were exclusively performed in models of post-natal development (rat, baboon and human) and the authors agreed to conclude that the extrapolation of red blood cell DHA level to that of the retina may be valid only during development, at the time when PUFA levels increase rapidly in neuronal tissues.

Because red blood cell fatty acid composition is a commonly used biomarker in human studies, the aim of our work was to check the validity of its use in adult humans for ocular tissues. For that purpose, we have investigated the possible associations between erythrocytes and retina/optic nerve lipids in tissues collected from human donors. The lipidomic analyzes were done through a double analytical approach, the standard and commonly-used gas chromatography methodology and the more specific liquid chromatography coupled to an electrospray ionization source-mass spectrometer (LC-ESI-MS).

## Methods

### Ethics Statement

Collection of the samples from human deceased subjects was conducted in accordance with the guidelines of the Declaration of Helsinki. A written consent was obtained and the protocol was accepted by the local ethics committee (CPP Sud Est I, CHU Saint Etienne, Saint Etienne, France).

### Human tissues

Human eyeballs and erythrocytes were obtained from 9 donors (bodies donated to science, 5 women and 4 men, mean age 84.3±8.5 years, range 72–97 years, **Table 1**), within 16 h (median time, mean time 15±7 h) after death. The bodies were refrigerated at +4°C in the first hours after death (less than 24 h). A blood sample was collected in heparinized tubes by venipuncture. Red blood cells were separated from plasma by centrifugation at 3000 rpm for 10 min at +4°C. Eyeballs were enucleated at the anatomy laboratory of the Saint Etienne School of Medicine (France). Enucleated eyeballs were immersed in a balanced saline solution (BSS, Alcon, Rueil Malmaison, France) at +4°C. Within 30 min after enucleation, a circular section at the pars plana was taken with a 18-mm diameter trephine and the corneoscleral disc was removed for other studies. The posterior pole of the eyeball was placed on a back-lit table and the retina was observed under an operating microscope to select the tissues further included in this study. No eye having large drusen, severe pigment epithelial alterations, severe macular atrophy, macular hemorrhage, or any grossly visible chorioretinal pathologic abnormality was included in the study. Moreover, a gross examination of the optic nerve head did not reveal any case of Glaucoma within the donors. The vitreous body was carefully removed. The entire neural retina (n = 9) was carefully separated from the RPE/choroid with forceps. The optic nerves (n = 6) were excised by cutting tangential to the sclera after having removed the extraocular tissues. All samples were immediately stored at −80°C until further analyses.

**Table 1:** Characteristics of the human donors, tissue collection, and sample analyzes.

subject	gender	age	post mortem delay before tissue collection	Collected tissues	LC-ESI-MS analyzes
#1	female	82	15.0	erythrocytes, retina	PC, PE
#2	male	97	9.5	erythrocytes, retina, optic nerve	PC, PE$
#3	male	72	24.0	erythrocytes, retina, optic nerve	PC, PE
#4	female	82	7.0	erythrocytes, retina, optic nerve	PC, PE
#5	female	88	6.5	erythrocytes, retina	PC
#6	male	90	14.0	erythrocytes, retina	PC
#7	male	75	21.0	erythrocytes, retina, optic nerve	PC
#8	female	94	23.5	erythrocytes, retina, optic nerve	PC
#9	female	79	24.0	erythrocytes, retina, optic nerve	PC, PE
**mean ± SD** *	-	84.3±*8.5*	16.1±*7.3*	-	-
**median**	-	82.0	15.0	-	-
**range**	-	72–97	6.5–24.0	-	-

* : Standard Deviation.

$ : no data for retinal PE for this subject.

### Lipid analyses

All chemical reagents were purchased from Sigma-Aldrich (St Quentin Fallavier, France), chloroform and methanol from SDS (Peypin, France). HPLC-grade N-hexane, chloroform and isopropanol, LC-MS-grade methanol and water were all from Fisher Scientific (Illkirch, France). All phospholipid standards were obtained from Avanti Polar Lipids Inc. (Alabaster, AL, USA).

#### Lipid extraction from human tissues

Lipids were extracted from the retinas and optic nerves following the Folch's procedure [35] whereas they were isolated from erythrocytes according to the method of Moilanen and Nikkari [36]. Phospholipids were purified from total lipid extracts using silica cartridges (25×10 mm i.d.; Sep-pack, Waters S.A., Framingham, MA, USA), as previously described by our group [31], [37]. Phospholipid extracts were stored under inert gas until further analyses.

#### Fatty acid analysis by gas chromatography

Total phospholipids from neural retina, optic nerve, and red blood cells were transmethylated using boron trifluoride in methanol according to Morrison and Smith [38]. Fatty acid methyl esters (FAMEs) and dimethylacetals (DMAs) were subsequently extracted with hexane and analyzed by gas chromatography on a Hewlett Packard Model 5890 gas chromatograph (Palo Alto, CA, USA) using a CPSIL-88 column (100 m×0.25 mm i.d., film thickness 0.20 µm; Varian, Les Ulis, France) equipped with a flame ionization detector. Hydrogen was used as the carrier gas (inlet pressure 210 kPa). The oven temperature was held at 60°C for 5 min, increased to 165°C at 15°C/min and held for 1 min, and then to 225°C at 2°C/min and finally held at 225°C for 17 min. The injector and the detector were maintained at 250°C. FAMEs and DMAs were identified by comparison with commercial and synthetic standards. The data were processed using the EZChrom Elite software (Agilent Technologies, Massy, France) and reported as a percentage of the total fatty acids.

#### Structural analysis of phospholipids by LC-ESI-MS

Prior to LC-ESI-MS analyses, the phosphorus content of the total phospholipid extract was determined according to the method developed by Bartlett and Lewis [39]. The total phospholipids were dried under nitrogen, and internal standards dimyristoyl-sn-glycerol-3-phosphatidylethanolamine (PE14:0/14:0, final concentration of 0.2 ng/µL) and dimyristoyl-sn-glycerol-3-phosphatidylcholine (PC14:0/14:0, final concentration of 0.4 ng/µL) were added. The samples were diluted at a final concentration of 12.5 ng/µL in chloroform/methanol (1∶1, v/v) for analysis and stored at −80°C under argon atmosphere.

The total phospholipids were analysed by liquid chromatography coupled to a mass spectrometer equipped with an ESI source. Liquid chromatography was performed using a Jasco PU 2089 Plus LC pump equipped with a Jasco AS 2057 Plus auto-sampler (Jasco Analytical Instruments, Bouguenais, France). The injection volume was of 5 µL. The separation was performed using a Hypersil Gold Silica Column (150 mm×2.1 mm i.d. ×3 µm, ThermoFinnigan, San Jose, CA, USA). The mobile phase consisted of (A) hexane/propan-2-ol/chloroform/water (44/43.5/10.5/2, v/v/v/v) containing 12.5 mM of ammonium formate and (B) hexane/propan-2-ol/chloroform/water (34/49/10.5/6.5, v/v/v/v) containing 12.5 mM of ammonium formate. The solvent-gradient system was as follows: 0 min A/B (%) 100/0, 10.5–24 min A/B (%) 22/78, 26.5–45 min A/B (%) 0/100% and 46–60 min A/B (%) 100/0. The flow rate was 300 µL.min^−1^ and the column was maintained at 30°C. The flow from LC was split using an analytical fixed flow splitter (split ratio = 1∶1, post-column, Analytical Scientific Instruments, El Sobrante, CA, USA). Mass spectrometry was performed using a ThermoFinnigan TSQ Quantum triple quadrupole mass spectrometer equipped with a standard electrospray ionisation source outfitted with a 100-µm i.d. H-ESI needle. The source spray head was oriented at a 90° angle orthogonal to the ion-transfer tube. Nitrogen was used for both the sheath and the auxiliary gases. The MS signals of phosphatidyl-choline species (PC) were first optimised by continuous infusion of the standards dissolved in the mobile phase using ESI in negative and positive modes. The electrospray ionisation spray voltages were 3 kV and −4.5 kV in negative and positive ion modes, respectively. The vaporiser temperature was of 150°C, sheath gas N_2_ pressure 45 (arbitrary units), auxiliary gas pressure 45 (arbitrary units), ion sweep gas pressure 5, ion transfer capillary temperature 300°C, skimmer offset 5 V and multiplier gain 300,000.

When operated under full scan conditions in the negative and positive ion modes, data were collected in the range of *m/z* 400–1100 amu with a scan time of 0.5 s. For PC characterization in the negative mode, ESI-MS/MS was used with argon as the collision gas at 1.5 mTor, and the collision energy was set to 15 eV; in the positive mode, the collision gas pressure was 0.8 mTor and the collision energy ranged from 30 to 45 eV. Phosphatidyl-ethanolamine (PE) and PC species were manually identified with the parent mass information and their characteristic fragment ions in the CID spectrum using lists of phospholipid species from our laboratory [31], [40].

Upon CID in the positive mode, PC and choline-plasmalogen species (plasmenyl-choline, PlsC) showed an intense fragment at *m/z* 184 amu due to their common choline head group. This fragment at *m/z* 184 amu was used for precursor ion scanning, whereby the [M+H]^+^ ions of PCs are specifically detected and quantified. For all calculations, the ratio of peak area of each PC species to the peak area of the internal standard (PC14:0/14:0) was used. Upon CID in the positive mode, [M+H]^+^ ions of PE loose their ethanolaminephosphate head group as a neutral fragment of 141 Da. Therefore, positive-ion mode neutral loss scanning of 141 Da was used for the selective detection and quantification of PE. For all calculations, the ratio of peak area of each PE species to the peak area of the internal standards (PE14:0/14:0) was used.

During the quantification step, a particular attention was paid to the influence of different parameters related to PE and PC acyl-chain length, degree of acyl-chain unsaturation on spectrometer response according to previous studies [40], [41], [42], [43]. Thus the relationship between the MS peak intensities of different PC species and their carbon chain length was included for calibration as described in our previous work [40]. Concerning PE species, similar precautions were taken in this quantitative study because different PE molecular species are not detected with equal efficiency response. Moreover, previous studies indicated that the total phospholipid concentration affect the quantification of PE species by ESI-MS/MS [44]. Therefore, we investigated the influence of these different parameters on the instrument response. First, to establish the range of linear response of our ESI-MS/MS instrument, an equimolar mixture was prepared with standards of PE14:0/14:0, 1,2-dipalmitoyl-*sn*-glycero-3-phosphocholine (PE16:0/16:0), 1-palmitoyl-2-oleoyl-*sn*-glycero-3-phosphoethanolamine (PE16:0/18:1), 1,2-distearoyl-*sn*-glycero-3-phosphoethanolamine (PE18:0/18:0), 1,2-dioleoyl-*sn*-glycero-3-phosphoethanolamine (PE18:1/18:1), 1,2-dilinoleoyl-*sn*-glycero-3-phosphoethanolamine (PE18:2/18:2), 1-stearoyl-2-arachidonoyl-*sn*-glycero-3-phosphoethanolamine (PE18:0/20:4), 1-stearoyl-2-docosahexaenoyl-*sn*-glycero-3-phosphoethanolamine (PE18:0/22:6), and 1,2-didocosahexaenoyl-*sn*-glycero-3-phosphoethanolamine (PE22:6/22:6) diluted from 10 pg/µL to 2 ng/µL per species (from 90 pg/µL to 18 ng/µL of total PE) in chloroform/methanol (1∶1) and then injected in the LC-ESI-MS instrument. The PE mixture had a linear response of ion count versus concentration in this range of concentration. Furthermore we have established a calibration curve between the intensity of the observed quasi-molecular ion species and the length and degree of unsaturation of the carbon chains. Linear response curves were obtained with increased concentrations of PE16:0/16:0, PE16:0/18:1, 1-stearoyl-2-linoleoyl-*sn*-glycero-3-phosphoethanolamine (PE18:0/18:2), PE18:0/18:0, PE18:0/20:4, PE18:0/22:6, 1,2-eicosanoyl-*sn*-glycero-3-phosphoethanolamine (PE20:0/20:0) and PE22:6/22:6 (range, 10 pg/µL to 2 ng/µL) and fixed concentrations of PE14/14:0 (0.2 ng/µL). Our results confirmed that the instrument response for PE species decreases when the phospholipid acyl chain length is increased. The responses remain a linear function of the acyl chain length, even at the highest concentrations. Thus, similarly to PC, a calibration function was then calculated and used to correct the experimental ion abundance in order to obtain the true molar abundance.

Since the use of the neutral loss of 141 Da is problematic in the quantification of ethanolamine-plasmalogen species (plasmenyl-ethanolamine, PlsE) [45], the method was based on multiple reaction monitoring (MRM) of one parent/fragment transition for each selected plasmalogen and PE14:0/14:0 as internal standard. For example, the MS^2^ fragmentation of the selected molecular ion [M-H]^−^ at *m/z* 728 amu of commercial 1-(1Z-octadecenyl)-2-oleoyl-*sn*-glycero-3-phosphoethanolamine (PlsE18:0/18:1) showed a principal fragment [R_2_CH_2_COO]^−^ resulting from the loss of the sn-2 acyl group: *m/z* 281 amu, which corresponds to oleic carboxylate. Thus, PlsE18:0/18:1 was quantified using the specific transition 728 → 281 amu. The method was then optimized on the specific transition parent/fragment of the two other commercial PlsE standards 1-(1Z-octadecenyl)-2-arachidonoyl-*sn*-glycero-3-phosphoethanolamine (PlsE180/20:4, 750 → 303 amu) and 1-(1Z-octadecenyl)-2-docosahexaenoyl-*sn*-glycero-3-phosphoethanolamine (PlsE180/22:6, 774 → 327 amu) in addition to PlsE18:0/18:1 (728→281 amu) and PE14:0/14:0 (634 → 227 amu). Linear response curves were obtained with increased concentrations of PlsE180/18:1, PlsE180/20:4 and PlsE180/22:6 (range 10 pg to 2 ng/µL per species) and fixed concentrations of PE14:0/14:0 (0.2 ng/µL). The standard curves were plotted as the ratio height of the respective compound/height of PE14:0/14:0 versus the concentration. Considering that all PlsE have the same basic chemical structure and differ only by different fatty acid side chains and that the principal fragment is the sn*-2* fatty acid, the theoretical LC-MS/MS parent fragments transitions of PlsE were determined. The data were processed using the Xcalibur software (ThermoFinnigan). Corrections were applied to the data for isotopic overlap.

### Statictical analyses

The data were analyzed using SAS software (SAS Institute, Cary, NC, USA). The non-parametric Spearman correlation coefficients were calculated to determine associations between tissue profiles. Associations were considered significant at a *P* level of less or equal to 0.05.

## Results

The characteristics of the human donors and of the studied samples are presented in **Table 1**. Nine human donors were included in this study. Five of them were females, and 4 were males. The median age of the donors was of 82 years (mean ± SD: 84.3±8.5 years). Erythrocytes and retinas were collected from all human donors whereas optic nerves were obtained from 6 subjects (3 females and 3 males). The median post-mortem delay for tissue collection was of 15 hours (mean ± SD: 16.1±7.3 hours), with a maximum time of 24 hours for two of the donors. Previous results from our laboratory have shown that even a mean post-mortem delay of 34 hours do not enhance the degradation of tissue lipids, as shown by the absence of free fatty acids and the lack of negative association between retinal DHA and post-mortem delay [46].

### Fatty acid compositions and associations emerging from standard gas chromatographic analyses

#### Fatty acid composition

**Figure 1:**
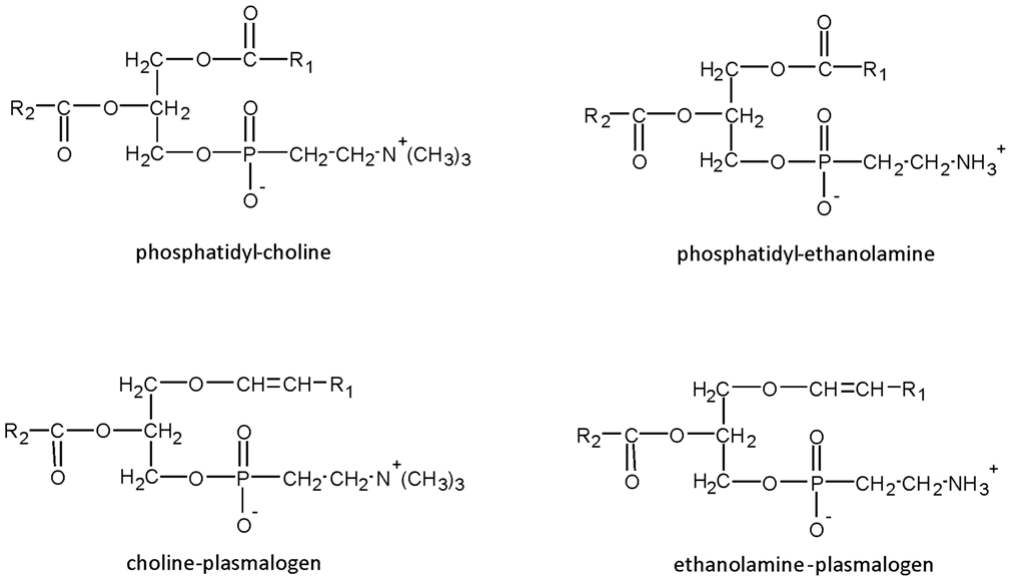
Structure of conventional phospholipids and plasmalogens. Conventional phospholipids such as phosphatidyl-choline and phosphatidyl-etanolamine contain ester bonds in order to link R1 and R2 acyl- moieties at the *sn*-1 and *sn*-2 positions of glycerol, respectively. As for ethanolamine- and choline- plasmalogens, they have a vinyl ether bond at the *sn*-1 position of the glycerol backbone to link alkenyl- moieties and an ester bond at the *sn*-2 position to link acyl- residues.

Gas chromatography gave an overview of the relative abundance of FAMEs and DMAs. FAMEs are formed by transmethylation of acyl- moieties issued from conventional phospholipids and plasmalogens whereas DMAs are from alkenyl- moieties issued from plasmalogens only (**Figure 1**) [38].

The complete fatty acid composition of erythrocytes, retinas and optic nerves obtained by standard gas chromatography is available in **Table 2**. It confirmed earlier observations on human retinas [32], [46], and erythrocytes [46]. To our knowledge, no previous data was available on the fatty composition of human optic nerve. The erythrocytes were characterized by high levels of palmitic acid (C16:0), stearic acid (C18:0), oleic acid (C18:1n-9), linoleic acid (C18:2n-6), and arachidonic acid (C20:4n-6). PUFAs from the omega-3 family were present at lower levels, the most abundant being DHA at a median level of 3.46% of total FAMEs + DMAs. Whereas C16:0, C18:0, C18:1n-9, and C20:4n-6 were also present at high levels in the retina, the amount of C18:2n-6 was lower (median value of 1.49% of total FAMEs + DMAs) and those of DHA were higher (median value of 15.03% of total FAMEs + DMAs) when compared to erythrocytes. As described previously [46], the inter-individual differences were low for the retina except for DHA (CV = 17.4%, range: 10.96–19.15% of total FAMEs + DMAs). The optic nerve was characterized by very high levels of C16:0, C18:0, and C18:1n-9 (median values of 14.87%, 13.90%, and 25.39%, of total FAMEs + DMAs, respectively). The levels of C20:4n-6 and DHA were low since their median values did not exceed 4.85% and 1.39% of total FAMEs + DMAs, respectively. The most specific feature of the optic nerve was its high content in DMAs when compared to the other tissues. Since the percentage of DMAs is known to reflect the half of the plasmalogen pool [47], plasmalogens account for about 36% of the phospholipids in the optic nerve whereas they represent about 9 to 10% in both erythrocytes and retinas.

**Table 2:** Fatty acid composition of total phospholipids purified from erythrocytes, retinas and optic nerves from human donors evaluated by gas chromatography (% of total fatty acids).

	erythrocyte				retina				optic nerve		
	*n = 9*			normal value/range [4], [23], [25], [32], [72], [77]	*n = 9*			normal value/range [4], [46]	*n = 6*		
	mean ± *SD*	median	range		mean ± *SD*	median	range		mean ± *SD*	median	range
C14:0	0.29±*0.27*	0.14	0.06–0.71	*0.3–0.7*	0.41±*0.06*	0.40	0.32–0.51	*0.4*	0.62±*0.15*	0.65	0.42–1.29
C15:0	0.11±*0.03*	0.11	0.08–0.17	*0.2–0.3*	0.11±*0.03*	0.11	0.07–0.16	*0.1*	0.14±*0.05*	0.14	0.09–0.23
DMA16:0^a^	2.07±*1.09*	1.98	0.49–3.90	*−*	1.38±*0.18*	0.31	1.17–1.66	*1.3*	4.55±*0.82*	4.53	3.23–5.32
C16:0	24.09±*5.29*	20.88	19.62–32.11	*16.2–31.0*	20.76±*1.29*	20.23	19.31–22.53	*18.8*	14.91±*1.73*	14.87	12.66–19.93
C16:1n-9	0.13±*0.03*	0.12	0.10–0.19	*0.6–0.8*	0.65±*0.06*	0.64	0.58–0.75	*0.9*	0.33±*0.06*	0.33	0.27–0.44
C16:1n-7	0.70±*0.22*	0.72	0.28–0.95	*−*	0.60±*0.18*	0.61	0.38–0.87	*0.8*	0.64±*0.11*	0.69	0.45–2.12
C17:0	0.34±*0.09*	0.38	0.16–0.46	*0.4–0.5*	0.24±*0.08*	0.29	0.12–0.31	*0.2*	0.31±*0.07*	0.30	0.25–0.42
DMA18:0^a^	2.16±*1.49*	1.74	0.42–4.13	*−*	2.61±*0.27*	2.67	2.29–3.04	*2.8*	3.12±*0.25*	3.00	2.00–3.39
DMA18:1n-9^a^	0.50±*0.32*	0.43	0.18–0.91	*−*	0.26±*0.04*	0.26	0.19–0.31	*0.3*	3.78±*0.43*	3.84	2.49–4.13
DMA18:1n-7^a^	0.19±*0.10*	0.15	0.05–0.38	*−*	0.47±*0.12*	0.45	0.32–0.71	*0.5*	5.81±*0.99*	5.78	4.32–7.04
C18:0	13.51±*0.87*	13.56	12.05–14.80	*11.5–17.0*	20.07±*0.89*	20.10	18.42–20.97	*19.4*	14.31±*1.14*	13.90	10.73–15.94
C18:1n-9	16.40±*2.29*	15.64	13.78–20.25	*13.4–18.3*	13.84±*1.77*	14.07	10.71–16.23	*14.3*	25.32±*0.54*	25.39	24.77–27.06
C18:1n-7	2.34±*0.88*	2.07	1.45–4.37	*−*	3.50±*0.51*	3.48	2.76–4.49	*3.4*	4.70±*0.43*	4.77	4.21–5.34
C18:2n-6	10.72±*1.64*	10.16	8.29–13.61	*9.7–12.6*	1.57±*0.27*	1.49	1.13–1.97	*1.7–2.5*	1.04±*0.21*	1.08	0.73–2.07
C20:0	0.24±*0.04*	0.24	0.18–0.28	*trace–0.1*	0.47±*0.05*	0.47	0.38–0.53	*0.6*	0.27±*0.07*	0.25	0.20–0.37
C18:3n-6	0.05±*0.03*	0.05	0.02–0.09	*trace–0.1*	0.16±*0.07*	0.14	0.09–0.28	*0.2*	*nd*	*nd*	*nd*
C20:1n-9	0.27±*0.08*	0.24	0.18–0.44	*0.1–0.2*	0.44±*0.10*	0.44	0.29–0.60	*0.6*	3.04±*0.32*	3.08	1.94–3.30
C18:3n-3	0.17±*0.07*	0.15	0.09–0.33	*trace–0.2*	0.10±*0.03*	0.11	0.05–0.13	*trace–0.02*	0.72±*0.12*	0.77	0.58–0.86
C20:2n-6	0.25±*0.05*	0.25	0.19–0.33	*0.1–0.4*	0.15±*0.03*	0.15	0.09–0.20	*0.2*	0.25±*0.03*	0.24	0.15–0.29
C20:3n-9	0.22±*0.19*	0.16	0.07–0.67	*0.1–2.0*	0.23±*0.13*	0.17	0.10–0.48	*0.4*	0.45±*0.10*	0.46	0.32–0.59
C22:0	0.61±*0.09*	0.60	0.52–0.77	*−*	0.33±*0.05*	0.31	0.27–0.39	*0.3*	0.18±*0.03*	0.18	0.15–0.22
C20:3n-6	1.40±*0.34*	1.39	0.90–1.81	*1.9–2.0*	1.68±*0.22*	1.67	1.25–2.06	*1.4–1.8*	0.57±*0.07*	0.55	0.42–0.64
C22:1n-9	0.06±*0.01*	0.07	0.05–0.07	*1.2*	0.07±*0.02*	0.07	0.04–0.09	*0.1*	0.30±*0.04*	0.29	0.23–0.35
C20:4n-6	11.64±*2.79*	12.80	7.39–14.30	*4.4–17.0*	10.98±*0.77*	11.16	10.01–12.37	*10.6–11.5*	4.71±*0.38*	4.85	4.20–5.19
C24:0	1.70±*0.39*	1.57	1.12–2.36	*−*	0.20±*0.09*	0.22	0.07–0.32	*0.3*	1.28±*0.15*	1.21	0.92–1.50
C20:5n-3	0.52±*0.18*	0.53	0.14–0.73	*0.1–1.0*	0.17±*0.08*	0.17	0.06–0.29	*0.1–0.2*	*nd*	*nd*	*nd*
C24:1n-9	2.06±*0.45*	1.96	1.48–2.88	*−*	0.10±*0.02*	0.11	0.07–0.14	*0.2*	2.37±*0.38*	2.19	1.64–2.85
C22:4n-6	1.95±*0.67*	2.12	0.90–2.87	*3.2–4.8*	1.41±*0.22*	1.36	1.17–1.88	*1.6–2.6*	4.00±*0.78*	3.63	2.60–5.09
C22:5n-6	0.48±*0.13*	0.45	0.35–0.73	*0.3–1.1*	0.75±*0.36*	0.70	0.35–1.45	*0.6–0.7*	0.36±*0.04*	0.34	0.32–0.41
C22:5n-3	1.43±*0.55*	1.53	0.46–2.03	*1.4–3.0*	0.96±*0.14*	0.93	0.77–1.16	*0.8–1.1*	0.31±*0.04*	0.31	0.27–0.36
C22:6n-3	3.12±*1.21*	3.46	1.35–5.00	*0.6–4.3*	15.34±*2.67*	15.03	10.96–19.15	*12.3–15.4*	1.47±*0.15*	1.39	1.15–1.68
Total DMA^a^	4.92±*1.91*	4.70	1.61–7.49	*−*	4.71±*0.50*	4.81	4.17–5.38	*−*	16.65±*2.39*	17.92	13.59–18.74
Total n-6	26.49±*3.93*	27.59	20.44–32.42	*19.3–30.6*	16.72±*0.96*	16.70	15.48–18.75	*18.0*	10.87±*0.57*	10.95	9.91–11.48
Total n-3	5.24±*1.79*	6.06	2.04–7.89	*5.9–6.9*	16.57±*2.62*	16.21	12.24–20.56	*13.2*	2.46±*0.20*	2.45	2.24–2.79
n-6/n-3	5.62±*2.10*	4.70	3.50–10.27	*−*	1.03±*0.18*	1.05	0.79–1.36	*−*	4.44±*0.40*	4.48	3.89–4.98
Total PUFA	31.95±*5.16*	34.69	23.10–36.87	*−*	33.51±*2.71*	32.97	29.02±37.48	*−*	13.78±*0.56*	13.88	13.05–14.59

a : corresponding to the derivative formed during methylation from alkenyl residues of plasmalogens; *nd*: not detected.

#### Associations between circulating and ocular concentrations

**Figure 2:**
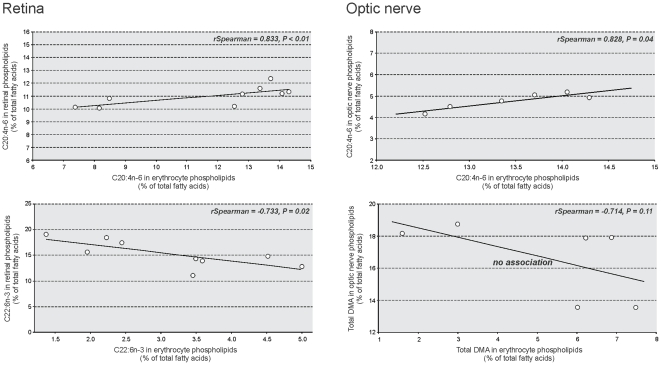
Selected significant associations between erythrocyte and retinal or optic nerve lipids after gas chromatographic analyses. Erythrocyte arachidonic acid (C20:4n-6) was positively associated with retinal C20:4n-6 (*rSpearman* = 0.833, *P*<0.01) and optic nerve C20:4n-6 (*rSpearma*n = 0.828, *P* = 0.04). Erythrocyte docosahexaenoic acid (C22:6n-3, DHA) was negatively associated with retinal DHA (*rSpearman* = −0.733, *P* = 0.02) whereas no significant association emerged between erythrocyte and optic nerve levels of plasmalogens (evaluated by dimethylacetals, DMA).

The complete list of statistically significant associations emerging from gas chromatographic analyzes between erythrocyte and retinal/optic nerve fatty acids is presented in **Table 3**. No association was found between erythrocyte DMAs and retinal DMAs. However, DMA18:1n-9 and DMA18:1n-7 in erythrocytes were positively associated with α-linolenic acid (C18:3n-3) and C20:4n-6 (*rSpearman* = 0.848, *P*<0.01 and *rSpearman* = 0.756, *P* = 0.02, respectively) and C20:4n-6 (*rSpearman* = 0.671, *P* = 0.04) in the retina, respectively. C20:4n-6 in erythrocytes was positively associated with retinal C20:4n-6 (*rSpearman* = 0.833, *P* = 0.02) (**Figure 2**), and negatively associated with DHA in the retina (*rSpearman* = −0.867, *P*<0.01). Retinal individual n-3 PUFAs were negatively associated not only with n-6 PUFAs but also with other individual n-3 PUFAs in erythrocytes. This was particularly true for retinal DHA, whose level was negatively related to those of n-6 docosapentaenoic acid (C22:5n-3) and C22:6n-3 in erythrocytes (*rSpearman* = −0.900, *P*<0.001 and *rSpearman* = −0.733, *P* = 0.02, respectively; **Figure 2**). No significant association was found between erythrocyte and retinal n-6/n-3 ratios (*rSpearman* = −0.493, *P* = 0.18).

**Table 3:** Statistically significant associations between erythrocyte and optic nerve or retinal phospholipid fatty acid compositions evaluated by gas chromatography.

erythrocyte	retina			optic nerve		
		*rSpearman*	*P*		*rSpearman*	*P*
DMA16:0^a^	*no association*			C20:4n-6	*0.863*	*0.02*
DMA18:0	*no association*			*no association*		
DMA18:1n-9	C18:3n-3C20:4n-6	*0.848* *0.756*	*<0.01* *0.02*	C20:4n-6	*0.840*	*0.03*
DMA18:1n-7	C20:4n-6	*0.671*	*0.04*	C18:2n-6	*0.771*	*0.04*
total DMA	*no association*			*no association*		
C18:2n-6	*no association*			*no association*		
C18:3n-6	*no association*			total n-3n-6/n-3	*−0.815* *1.000*	*0.01* *<0.0001*
C20:2n-6	*no association*			C22:4n-6	*0.943*	*<0.01*
C20:3n-6	C20:5n-3	*−0.740*	*0.02*	C22:6n-3total n-3	*−0.985* *−0.927*	*<0.01* *<0.01*
C20:4n-6	C20:4n-6C22:6n-3	*0.833* *−0.867*	*<0.01* *<0.01*	C20:4n-6	*0.828*	*0.04*
C22:4n-6	C22:6n-3	*−0.667*	*0.04*	*no association*		
C22:5n-6	*no association*			*no association*		
total n-6	total n-3n-6/n-3	*−0.658* *0.616*	*0.05* *0.04*	*no association*		
C18:3n-3	*no association*			*no association*		
C20:5n-3	*no association*			C22:5n-6	*−0.880*	*0.02*
C22:5n-3	C22:6n-3	*−0.900*	*<0.001*	*no association*		
C22:6n-3	C22:6n-3	*−0.733*	*0.02*	*no association*		
total n-3	total PUFA	*−0.883*	*<0.01*	*no association*		
n-6/n-3	total PUFA	*0.867*	*<0.01*	*no association*		
total PUFA	*no association*			*no association*		

a : corresponding to the derivative formed during methylation from alkenyl- residues of plasmalogens.

As observed for the retina, no association was found between optic nerve DMAs and erythrocyte DMAs (**Figure 2**). Erythrocyte DMA16:0 and DMA18:1n-9 were positively associated with optic nerve C20:4n-6 (*rSpearman* = 0.863, *P* = 0.02 and *rSpearman* = 0.840, *P* = 0.03, respectively). Erythrocyte DMA18:1n-7 was positively associated with optic nerve C18:2n-6 (*rSpearman* = 0.771, *P* = 0.04). The remaining statistically significant associations between erythrocytes and optic nerve concerned almost exclusively n-6 PUFAs in erythrocytes. These ones were either positively associated with n-6 PUFAs, or negatively associated with n-3 PUFAs in the optic nerve. Indeed, C20:4n-6 and eicosadienoic acid (C20:2n-6) in erythrocytes were positively associated with C20:4n-6 and n-6 docosatetraenoic acid (C22:4n-6) in the optic nerve, respectively (*rSpearman* = 0.828, *P* = 0.04 and *rSpearman* = 0.943, *P*<0.01, respectively) (**Figure 2**) whereas C18:3n-6 and n-6 eicosatrienoic acid (C20:3n-6) in erythrocytes were negatively associated with total n-3 PUFAs and DHA in the optic nerve. No significant association was found between C20:4n-6 in erythrocytes and DHA in the optic nerve.

### Concentrations of individual phospholipid species and associations emerging from LC-ESI-MS analyzes

#### Separation of phospholipids by LC

**Figure 3:**
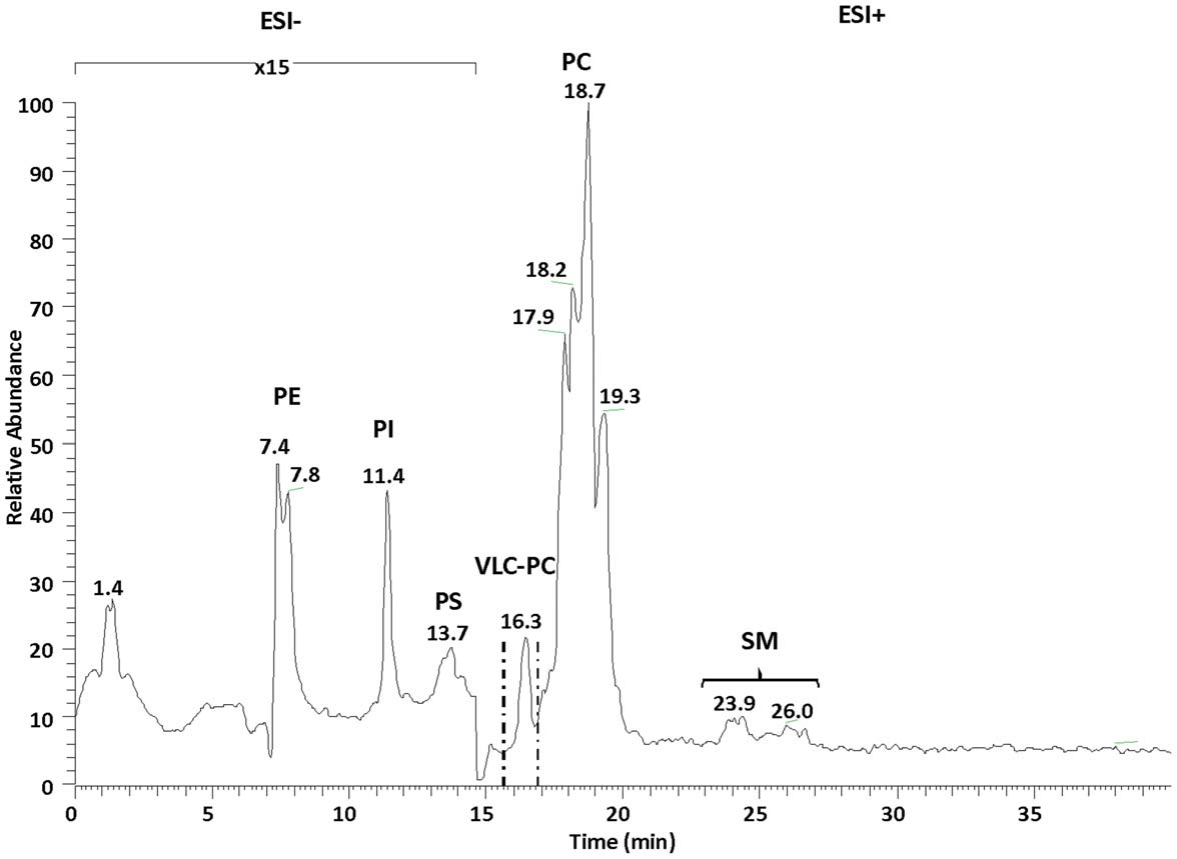
LC-ESI-MS normal-phase chromatogram of the lipid extract from human retina. The retention times of phosphatidyl-ethanolamine (PE), phosphatidyl-inositol (PI), phosphatidyl-serine (PS), phosphatidyl-choline (PC), sphingomyelin (SM), and lyso-phosphatidyl-choline (LPC) classes were of 7–8.5 min, 11–12 min, 12–14 min, 15.5–22 min, and 23–27.5 min, respectively. The mass spectrometer was operated under full scan in the negative ion mode from 0 to 15 min and in the positive ion mode from 15 min to 40 min.

We have chosen to separate the phospholipid species using LC prior to the ESI-MS/MS analysis in order to enhance the detection of the minor isobaric species in the mixture. During the development of LC separation of total phospholipids, the mass spectrometer was operated under full scan conditions in the negative ion mode for detection of PE, phosphatidyl-inositol (PI), phosphatidyl-serine (PS) and lyso-phosphatidyl-ethanolamine (LPE) and under full scan conditions in the positive ion mode for quantification of PC, lyso-phosphatidyl-choline (LPC) and sphingomyelin (SM) species [40]. Successful separation of PE, PI, PS, PC, LPC, and SM was achieved using a silica gel column and a gradient of hexane/isopropanol/water containing ammonium formate as a mobile phase. Individual phospholipid classes were identified by comparing their retention volume and mass spectra to those of their corresponding standards (**Figure 3**). As expected, PlsE and PlsC species coeluted with PE and PC species, respectively. Under our HPLC conditions, PC species containing very long chain polyunsaturated fatty acids (VLC-PUFAs) eluted separately just prior to the other PC molecular species in the retina.

#### Characterization and quantification of PE, PlsE, PC and PlsC species

**Figure 4:**
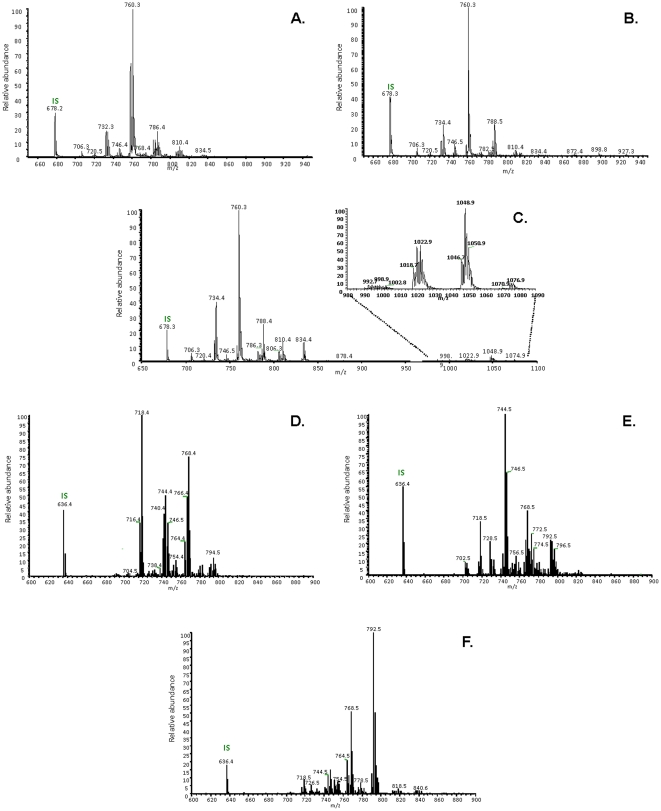
Positive-ion HPLC-ESI-MS Mass spectra of total phosphatidyl-choline fraction collected from human neural retina. A.) optic nerve (B.) and red blood cells (C.), by scanning for precursors at *m/z* 184 amu in the positive mode. Positive-ion HPLC-ESI-MS Mass spectra of total PE fraction collected from human neural retina D.), optic nerve (E.) and red blood cells (F.), using neutral loss scan at 141 amu in the positive mode.

A complete structural characterization of intact PE, PlsE, PC, and PlsC species including plasmalogens was obtained from human neural retina, optic nerve and red blood cells using electrospray tandem mass spectrometer (ESI-MS/MS) by collision-induced dissociation (CID) in the negative mode. Fatty acid composition and distribution have been clearly assigned based on the intensity of *sn*-2/*sn*-1 fragment ions as **Figure 4** shows HPLC-ESI-MS mass spectra of total PC and PE fractions after the addition of the internal standard.

The complete composition in individual species of PC and PlsC in erythrocytes, retinas and optic nerves is available in **Table S1**. Briefly, total choline-phospholipids (PC+PlsC) represented about 375, 490 and 390 µg per milligram of total phospholipids in erythrocytes, retinas, and optic nerve, respectively, thus confirming earlier observations having estimated these levels to be of 36%, 48%, and 40%, respectively [5], [7], [32]. The most represented species in all of the studied tissues was PC16:0/18:1. The retina was characterized by the presence of species with fatty acids having more than 30 carbon atoms, so called “very-long-chain PUFA” (VLC-PUFA), whereas the optic nerve contained species with VLC-PUFAs having 24 to 26 carbon atoms.

**Table S2** describes the concentrations in all individual species of PE and PlsE in erythrocytes, retinas and optic nerves. The erythrocyte content in PE was of 145 µg per milligram of total phospholipids, which is in agreement with the literature having estimated it to be of 19% [32]. So was that of the optic nerve with a mean concentration of 141 µg per milligram of total phospholipids when compared to previous observations having described values of 8.7% and 11.7% in samples from rabbit and bovine origin, respectively [5], [6]. The retinal content in PE showed more variability as confirmed by the large range of concentrations (from 292 to 608 µg per mg of total phospholipids). This may explain the higher mean value we have measured (400 µg per mg of total phospholipids) when compared to previous data from us or others showing that total PE species represent 29.5% and 31.7% of total phospholipids in bovine and human retinas, respectively [7], [47]. Within the three tissues studied, the retina was characterized by its high content in PE species esterified with DHA, representing almost 65% by mean of total PE species.

#### Associations between circulating and ocular concentrations of PC and PlsC

**Figure 5:**
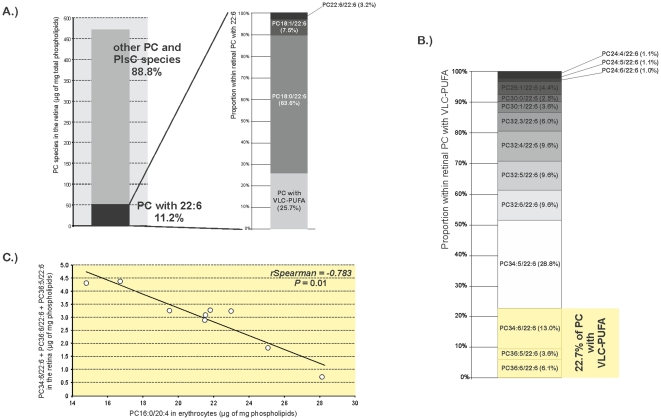
Erythrocyte PC16:0/20:4 as a possible marker of a pool of retinal VLC-PUFA. **A**): In the retina, VLC-PUFA accounted for about 25% of retinal PC species esterified to DHA, themself representing 11% of retinal total PC and PlsC. **B**): PC34:6/22:6, PC36:6/22:6, and PC36:5/22:6 were the longest and the most unsaturated VLC-PUFA in the retina. These three species accounted for 22.7% of total retinal VLC-PUFA. **C**) This pool of retinal VLC-PUFA was negatively associated with erythrocyte PC16:0/20:4 (*rSpearman* = −0.783, *P* = 0.01). Abbreviations of individual PC species are as follows: position on the glycerol backbone as shown as *sn-1*/*sn*-2 of the fatty alcohol radicals (abbreviated as *number of carbons: number of double bonds*).

The complete list of statistically significant associations between erythrocyte and retinal PC and PlsC is presented in **Table 4**. PC species with saturated or monounsaturated fatty acids in erythrocytes (PC14:0/16:0 and PC16:0/16:0) were negatively associated with other species with saturated and/or monounsaturated fatty acids in the retina. Other erythrocyte PC species (namely PC18:0/22:6 and PC20:6/22:6) were positively associated with other PC species containing DHA in the retina (PC18:1/22:6, PC22:6/22:6, and PC24:5/22:6). Finally, the most interesting result concerned PC16:0/20:4 in erythrocytes, which was negatively associated with PC species containing together DHA and VLC-PUFAs having more than 30 carbon atoms (PC30:0/22:6, *rSpearman* = −0.650, *P* = 0.05; PC34:6/22:6, *rSpearman* = −0.660, *P* = 0.05; PC36:6/22:6, *rSpearman* = −0.716, *P* = 0.02, and PC36:5/22:6, *rSpearman* = −0.717, *P* = 0.02). The retinal species containing DHA concerned only PC but not PlsC species. DHA-rich PC represented 11.2% of total choline-phospholipids and were esterified or not with VLC-PUFAs (**Figure 5A**). PC having VLC-PUFAs accounted for of 25.7% total PC with DHA. Fourteen PC species containing VLC-PUFAs were identified and quantified in the retina (**Figure 5B**). The longest and most unsaturated ones, namely PC34:6/22:6, PC36:6/22:6, and PC36:5/22:6, accounted for 22.7% of retinal VLC-PUFAs. Taken together, these three entities were negatively associated with PC16:0/20:4 in erythrocytes from human donors (*rSpearman* = −0.783, *P* = 0.01, **Figure 5C**).

**Table 4:** Statistically significant associations between erythrocyte and retinal individual choline-phospholipid concentrations evaluated by liquid chromatography coupled with tandem mass spectrometry.

erythrocytes			retina				
molecular species	[M+H]^+^	range of concentration*(µg of mg phospholipids)*	molecular species	[M+H]^+^	range of concentration*(µg of mg phospholipids)*	*rSpearman*	*P*
PC14:0/16:0^a^	706.50	1.36–3.91	PC16:0/18:0	762.59	21.33–27.88	*−0.610*	*0.05*
			PC18:1/18:1	786.59	8.56–15.16	*−0.736*	*0.02*
PC16:0/16:0	734.56	6.15–25.48	PC16:1/16:1	730.53	1.08–2.29	*−0.633*	*0.05*
			PC16:0/18:1	760.58	147.15–175.04	*−0.697*	*0.02*
			PC16:0/18:0	762.59	21.33–27.88	*−0.626*	*0.05*
			PC18:1/18:1	786.59	8.56–15.16	*−0.754*	*0.01*
			PC18:0/18:1	788.61	29.79–44.54	*−0.713*	*0.02*
PC14:0/22:5	780.50	1.00–5.88	PC16:0/22:5	808.58	6.72–10.07	*0.724*	*0.01*
			PC36:5/22:6	1076.90	0.18–0.88	*0.636*	*0.04*
PC16:0/20:4	782.56	14.79–28.12	PC30:0/22:6	1002.70	0.05–0.50	*−0.650*	*0.05*
			PC34:6/22:6	1046.80	0.06–3.02	*−0.660*	*0.05*
			PC36:6/22:6	1074.80	0.38–1.58	*−0.716*	*0.02*
			PC36:5/22:6	1076.90	0.18–0.88	*−0.717*	*0.02*
PC18:1/18:2	784.58	7.42–16.10	PC16:0/20:4	782.56	9.69–14.74	*0.634*	*0.04*
			PC18:0/22:5	836.61	4.69–8.93	*0.733*	*0.02*
PC18:1/18:1	786.59	19.22–34.49	PC16:0/18:1	760.58	147.15–175.04	*−0.904*	*<0.01*
			PC18:1/18:1	786.59	8.56–15.16	*−0.833*	*<0.01*
PlsC18:1/20:2	796.61	1.47–2.56	PlsC16:0/16:1	720.00	1.32–2.67	*0.826*	*<0.01*
PC18:2/20:4 + PC16:0/22:6	806.56	3.42–15.62	PC22:6/22:6	878.56	0.60–3.81	*0.683*	*0.04*
PC18:0/20:3	812.61	1.90–4.59	PC18:0/22:5	836.61	4.69–8.93	*0.733*	*0.02*
PC18:0/22:6	834.59	1.36–5.67	PC18:1/22:6	832.58	3.41–4.51	*0.666*	*0.04*
			PC22:6/22:6	878.56	0.60–3.81	*0.738*	*0.01*
PC20:6/22:6	850.50	0.09–0.75	PC24:5/22:6	908.60	<0.01–0.39	*0.731*	*0.01*

a : Abbreviations of individual PC and PlsC species are as follows: position on the glycerol backbone as shown as sn-1/sn-2 of the fatty acid and fatty alcohol radicals (abbreviated as number of carbons: number of double bonds).

The complete inventory of statistically significant associations emerging from LC-ESI-MS analyzes of erythrocyte and optic nerve PC and PlsC is presented in **Table 5**. Within the 13 statistically significant associations found, 8 concerned plasmalogen species. Six of these entities were PlsC species with saturated, monounsaturated or polyunsaturated with 2 or three double bonds fatty acids in erythrocytes (namely PlsC16:0/16:1, PlsC18:1/18:0, PlsC18:0/18:0, PlsC18:1/20:2, PlsC18:0/20:3, and PlsC18:1/22:1) that were positively associated with other PlsC species having saturated or monounsaturated fatty acids in optic nerve. The remaining species were PlsC18:0/22:4 and PlsC18:1/22:4 whose concentrations in erythrocytes were positively associated to other PlsC species in the optic nerve. Concerning the concentrations of non-plasmalogen phospholipids in erythrocytes, they were negatively associated to those from the optic nerve. Within the several species found to display statistically significant associations, PC18:0/20:2 was particularly interesting since it was negatively associated with several PC species having VLC-PUFA with 22 to 25 carbon atoms and DHA at *sn*-1 and *sn*-2 positions of glycerol, respectively (PC22:3/22:6, *rSpearman* = −0.767, *P* = 0.04; PC22:2/22:6, *rSpearman* = −0.822, *P* = 0.02; PC24:3/22:6, *rSpearman* = −0.942, *P*<0.01, PC25:6/22:6, *rSpearman* = −0.837, *P* = 0.01; and PC25:3/22:6, *rSpearman* = −0.942, *P*<0.01).

**Table 5:** Statistically significant associations between erythrocyte and optic nerve individual choline-phospholipid concentrations evaluated by liquid chromatography coupled with tandem mass spectrometry.

erythrocyte			optic nerve				
molecular species	[M+H]+	range of concentration*(µg of mg phospholipids)*	molecular species	[M+H]+	range of concentration*(µg of mg phospholipids)*	*rSpearman*	*P*
PlsC16:0/16:1^a^	720.00	1.24–3.48	PlsC18:1/22:1	826.25	0.10–0.24	*0.942*	*<0.01*
PlsC18:1/18:0	772.61	2.74–4.65	PlsC18:1/22:1	826.25	0.10–0.24	*0.755*	*0.01*
PlsC18:0/18:0	774.63	2.44–3.99	PlsC18:1/22:1	826.25	0.10–0.24	*0.742*	*0.05*
PlsC18:1/20:2	796.61	1.47–2.56	PlsC18:1/18:0	772.61	1.48–3.88	*0.942*	*<0.01*
PC18:0/20:3	812.61	1.90–4.59	PC24:3/22:6	912.60	0.20–1.18	*−0.773*	*0.04*
			PC25:3/22:6	926.70	0.89–2.52	*−0.788*	*0.03*
PC18:0/20:2	814.60	0.48–1.71	PC22:3/22:6	884.60	0.28–1.15	*−0.767*	*0.04*
			PC22:2/22:6	886.60	0.30–0.76	*−0.822*	*0.02*
			PC24:3/22:6	912.60	0.20–1.18	*−0.942*	*<0.01*
			PC25:6/22:6	920.60	0.22–1.22	*−0.837*	*0.01*
			PC25:3/22:6	926.70	0.89–2.52	*−0.942*	*<0.01*
PlsC18:1/22:4	820.55	0.37–0.80	PlsC18:0/16:1+PlsC16:0/18:1+PlsC18:1/16:0	744.58	1.78–3.22	*0.885*	*0.01*
PlsC18:0/22:4	822.55	0.28–0.90	PlsC18:0/16:1+PlsC16:0/18:1+PlsC18:1/16:0	744.58	1.78–3.22	*0.828*	*0.04*
			PlsC18:1/18:0	772.61	1.48–3.88	*0.788*	*0.03*
			PlsC18:1/22:4	820.55	0.12–0.35	*0.828*	*0.04*
			PlsC18:1/22:1	826.25	0.10–0.24	*0.828*	*0.04*
PlsC18:1/22:1	826.25	0.08–0.47	PlsC18:1/18:0	772.61	1.48–3.88	*0.750*	*0.05*
			PlsC18:1/22:4	820.55	0.12–0.35	*0.942*	*<0.01*
			PlsC18:1/22:1	826.25	0.10–0.24	*0.942*	*<0.01*
PC18:0/22:5	836.61	0.97–1.76	PC24:3/22:6	912.60	0.20–1.18	*0.763*	*0.04*
			PC25:3/22:6	926.70	0.89–2.52	*0.751*	*0.05*
PC18:0/22:4	838.62	0.36–1.44	PC26:1/22:6	944.70	0.34–0.67	*−0.885*	*0.01*
PC20:3/22:6+PC20:4/22:5+PC20:5/22:4	856.60	0.04–0.71	PC18:0/22:5	836.61	0.56–1.43	*0.784*	*0.03*
			PC24:3/22:6	912.60	0.20–1.18	*0.847*	*0.01*
			PC25:6/22:6	920.60	0.22–1.22	*0.942*	*<0.01*
PC22:6/22:6	878.56	0.11–0.75	PC25:6/22:6	920.60	0.22–1.22	*0.828*	*0.04*

a : Abbreviations of individual PC and PlsC species are as follows: position on the glycerol backbone as shown as sn-1/sn-2 of the fatty acid and fatty alcohol radicals (abbreviated as number of carbons: number of double bonds).

#### Associations between circulating and ocular concentrations of PE and PlsE

**Figure 6:**
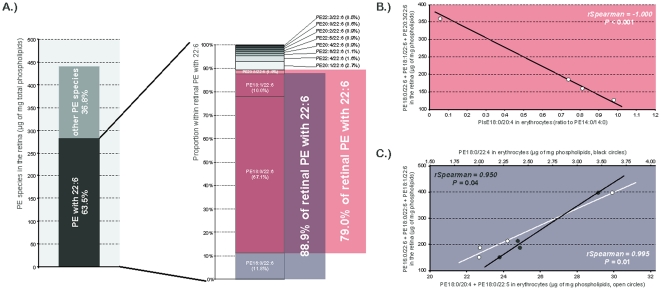
Identification of circulating indexes of retinal PE esterified to DHA. **A**) PE with DHA accounted for 63% of total retinal PE species. Within these entities, two different pools of molecules were of concern as they represented 79% and 89% of the total PE with 22:6. **B**) PlsE18:0/20:4 in erythrocytes was negatively associated to the first group represented by PE18:0/22:6+PE18:1/22:6+PE20:3/22:6 (*rSpearman* = −1.000, *P*<0.001). **C**) The second fraction of retinal PE with 22:6 represented by PE16:0/22:6+PE18:0/22:6+PE18:1/22:6 was positively associated to PE18:0/22:4 (black circles, *rSpearman* = 0.950, *P* = 0.04) and PE18:0/20:4+PE18:0/22:5 (open circles, *rSpearman* = −0.995, *P* = 0.01) in erythrocytes. Abbreviations of individual PE species are as follows: position on the glycerol backbone as shown as *sn-1*/*sn*-2 of the fatty alcohol radicals (abbreviated as *number of carbons: number of double bonds*).

The inventory of statistically significant associations between erythrocyte and retinal PE and PlsE is presented in **Table 6**. As for PC species, PE species with saturated or monounsaturated fatty acids in erythrocytes (PE16:0/16:0 and PC18:0/18:1) were positively associated with other species with saturated and/or monounsaturated fatty acids in the retina. Other PC entities having saturated or monounsaturated fatty acids at *sn*-1 and polyunsaturated fatty acid at *sn*-2 (namely PE16:0/18:2, PE18:0/18:2, and PE20:1/22:6) were positively associated with PE species esterified to DHA in the retina. PlsE18:0/20:4 was interesting since its concentrations in erythrocytes were negatively associated with those of three major retinal PE species esterified to DHA, namely PE20:3/22:6 (*rSpearman* = −0.991, *P*<0.01), PE18:0/22:6 (*rSpearman* = −0.973, *P* = 0.03), and PE18:1/22:6 (*rSpearman* = −0.977, *P* = 0.02). Taken together, these three retinal PE species represent about 79% of total retinal PE species with DHA, themselves accounting for 63.5% of total retinal PE (**Figure 6A**). When considering these three species together as a “pool of retinal DHA”, the negative association with erythrocyte PlsE18:0/20:4 was still significant (*rSpearman* = −1.000, *P*<0.0001 **Figure 6B**).By the same way, the concentrations of other PE entities in erythrocyte were associated to another subsequent pool of DHA-containing PE species. Indeed, the erythrocyte levels of PE18:0/22:4 were positively associated with those of retinal concentrations of PE16:0/22:6 (*rSpearman* = 0.972, *P* = 0.03), PE18:1/22:6 (*rSpearman* = 0.977, *P* = 0.02), and PE18:0/22:6 (*rSpearman* = 0.978, *P* = 0.02), this group representing 88.9% of total PE species with DHA. The positive association between erythrocyte PE18:0/22:4 and this group was also significant when these three entities were considered together (*rSpearman* = 0.950, *P* = 0.04 **Figure 6C**). The same pool of DHA-rich retinal PE (namely PE16:0/22:6, PE18:0/22:6, and PE18:1/22:6) was also positively associated with erythrocyte PE18:0/20:4 and PE18:0/22:5, when considered together (*rSpearman* = −0.995, *P* = 0.01 **Figure 6C**).

**Table 6:** Statistically significant associations between erythrocyte and retinal individual ethanolamine-phospholipid concentrations evaluated by liquid chromatography coupled with tandem mass spectrometry.

erythrocyte			retina				
molecular species	[M+H]+ or MS/MS transition^a^	range of concentration*(µg of mg phospholipids or ratio to PE14:0/14:0)* b	molecular species	[M+H]+	range of concentration*(µg of mg phospholipids or ratio to PE14:0/14:0)* a	*rSpearman*	*P*
PE16:0/16:0^c^	692.54	0.16–0.55	PE16:0/16:0	692.54	0.03–0.43	*0.948*	*0.05*
			PE 18:0/18:1	746.59	9.64–19.23	*0.948*	*0.05*
PE16:0/18:2	716.54	6.24–9.92	PE 16:0/22:6	764.54	20.22–50.68	*0.953*	*0.04*
			PE 18:0/22:6	792.57	116.65–294.27	*0.948*	*0.05*
PE18:0/18:2	744.57	10.03–13.93	PE 18:1/22:6	790.56	12.83–53.08	*0.981*	*0.02*
			PE 18:0/22:6	792.57	116.65–294.27	*0.982*	*0.02*
PE18:0/18:1	746.59	4.77–8.14	PE 16:0/16:0	692.54	0.03–0.43	*0.947*	*0.05*
			PE 18:0/18:1	746.59	0.64–19.23	*0.956*	*0.04*
PE18:0/22:4	796.60	2.19–3.42	PE 16:0/22:6	764.54	20.22–50.68	*0.972*	*0.03*
			PE 18:1/22:6	790.56	12.83–53.08	*0.977*	*0.02*
			PE 18:0/22:6	792.57	116.65–294.27	*0.978*	*0.02*
PE18:0/20:4	768.57	19.30–24.47	PE 16:0/22:6	764.54	20.22–50.68	*0.948*	*0.05*
PE18:0/22:5	794.59	3.01–5.21	PE 18:1/22:6	790.56	12.83–53.08	*0.990*	*0.01*
			PE 18:0/22:6	792.57	116.65–294.27	*0.993*	*<0.01*
PE20:1/22:6	818.56	<0.01–0.84	PE 16:0/22:6	764.54	20.22–50.68	*0.978*	*0.02*
			PE 18:1/22:6	790.56	12.83–53.08	*0.994*	*<0.01*
PlsE18:0/20:4	750->303	0.05–0.98	PE18:1/22:6	790.56	12.83–53.08	*−0.977*	*0.02*
			PE18:0/22:6	792.57	116.65–294.27	*−0.973*	*0.03*
			PE20:3/22:6	814.56	1.33–11.50	*−0.992*	*<0.01*
Total PlsE	-	0.33–6.24	Total PlsE	-	1.59–2.15	*0.953*	*0.04*

a : [M+H]+ for PE species and MS/MS transition for PlsE species.

b : PE species are expressed as µg of mg of total phospholipids whereas PlsE species are expressed as ratio to the internal standard PE14:0/14:0.

c : Abbreviations of individual PE and PlsE species are as follows: position on the glycerol backbone as shown as sn-1/sn-2 of the fatty acid and fatty alcohol radicals (abbreviated as number of carbons: number of double bonds).

**Figure 7:**
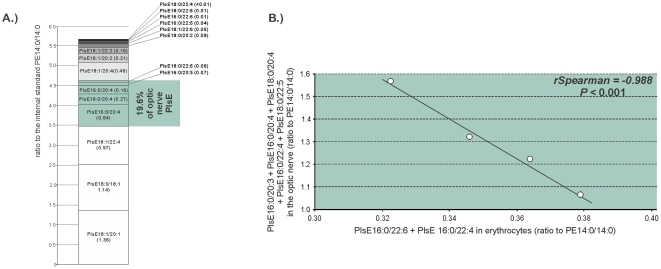
PlsE16:0/22:6 and PlsE16:0/22:4 as indexes of a pool of optic nerve PlsE. **A**) PlsE18:0/22:5, PlsE16:0/20:3, PlsE16:0/20:4, PlsE18:0/20:4, and PlsE16:0/22:4 represented 19% of optic nerve PlsE. B) This group of optic nerve PlsE was negatively associated to PlsE16:0/22:6 and PlsE16:0/22:4 in erythrocytes (*rSpearman* = −0.988, *P*<0.001). Abbreviations of individual PlsE species are as follows: position on the glycerol backbone as shown as *sn-1*/*sn*-2 of the fatty alcohol radicals (abbreviated as *number of carbons: number of double bonds*).

The list of statistically significant associations emerging from LC-ESI-MS analyzes of erythrocyte and optic nerve PE and PlsE is presented in **Table 7**. One PE specie esterified with DHA in erythrocytes were positively associated with other PE species with DHA in the optic nerve (PE18:0/22:6 with PE18:1/22:6, *rSpearman* = 0.948, *P* = 0.04). The circulating amounts of PE18:1/22:4 and/or PE16:0/22:5 were positively associated with the optic nerve concentrations of several PE species esterified with monounsaturated or polyunsaturated fatty acids (PE16:0/16:1, *rSpearman* = 0.988, *P* = 0.01; PE16:0/20:4, *rSpearman* = 0.950, *P* = 0.05; PE18:0/20:4, *rSpearman* = 0.950, *P* = 0.04; and PE18:1/22:6, *rSpearman* = 0.978, *P* = 0.02). The concentrations of the other PE species in erythrocytes (PE18:1/18:1 and/or PE18:0/18:2, PE18:1/20:4 and/or PE16:0/22:5, PE18:0/20:4, and PE20:0/20:3 and/or PE22:1/18:2) were negatively associated with PlsE species in the optic nerve (PlsE16:0/20:4, PlsE18:1/20:4, PlsE18:0/22:5, PlsE18:1/22:4, and PlsE18:0/22:6). Two circulating PlsE species, namely PlsE16:0/22:4 and PlsE16:0/22:6, were associated with six species of PlsE in the optic nerve. When considering the major five of them, namely PlsE16:0/20:3, PlsE16:0/20:4, PlsE18:0/20:4, PlsE16:0/22:4, and PlsE18:0/22:5, they represent almost 20% of the total optic nerve PlsE (**Figure 7A**). This pool of optic nerve PlsE was negatively associated with erythrocyte PlsE16:0/22:4 and PlsE16:0/22:6 considered together (*rSpearman* = 0.988, *P*<0.001.

**Table 7:** Statistically significant associations between erythrocyte and optic nerve individual ethanolamine-phospholipid concentrations evaluated by liquid chromatography coupled with tandem mass spectrometry.

erythrocyte			optic nerve				
molecular species	[M+H]+ or MS/MS transition^a^	range of concentration*(µg of mg phospholipids or ratio to PE14:0/14:0)* b	molecular species	[M+H]+ or MS/MS transition^a^	range of concentration*(µg of mg phospholipids or ratio to PE14:0/14:0)* b	*rSpearman*	*P*
PE18:1/18:1+PE18:0/18:2^c^	744.57	10.02–13.93	PlsE16:0/20:4	722 ->303	0.10–0.31	*−0.666*	*<0.01*
			PlsE18:1/20:4	748 ->303	0.32–0.54	*−0.998*	*<0.01*
PE18:1/20:4+PE16:0/22:5	766.56	14.10–20.24	PE16:0/16:1	690.53	0.09–0.83	*0.988*	*0.01*
			PE16:0/20:4	740.54	1.01–2.64	*0.950*	*0.05*
			PE18:0/20:4	765.57	14.15–20.76	*0.950*	*0.04*
			PE18:1/22:6	790.56	1.57–2.76	*0.978*	*0.02*
			PlsE18:0/22:5	776 ->329	0.05–0.06	*−0.974*	*0.02*
PE18:0/20:4	768.57	19.30–24.47	PlsE18:1/22:4	776 ->331	0.74–1.11	*−0.943*	*0.05*
PE18:0/22:6	792.57	2.36–7.47	PE18:1/22:6	790.56	1.57–2.76	*0.948*	*0.04*
PE20:0/20:3+PE22:1/18:2	798.62	<0.01–0.13	PlsE18:0/22:6	774 ->327	0.04–0.05	*−0.944*	*0.05*
			PlsE18:0/22:5	776 ->329	0.05–0.06	*−0.953*	*0.04*
PlsE16:0/22:6	746 ->327	<0.01–0.02	PlsE16:0/20:3	724 ->305	0.05–0.09	*0.874*	*0.05*
			PlsE16:0/22:4	750 ->331	0.39–0.59	*0.930*	*0.02*
PlsE16:0/22:4	750 ->331	<0.01–0.36	PlsE16:0/20:4	722 ->303	0.10–0.31	*−0.999*	*<0.01*
			PlsE18:1/20:4	748 ->303	0.32–0.54	*0.999*	*<0.01*
			PlsE18:1/22:6	772 ->327	<0.01–0.01	*−0.988*	*0.01*
			PlsE18:0/22:5	776 ->329	0.05–0.06	*0.970*	*0.03*
PlsE18:0/20:4	750 ->303	0.05–0.98	PE18:1/22:6	790.56	1.57–2.76	*0.962*	*0.03*

a : [M+H]+ for PE species and MS/MS transition for PlsE species.

b : PE species are expressed as µg of mg of total phospholipids whereas PlsE species are expressed as ratio to the internal standard PE14:0/14:0.

c : Abbreviations of individual PE and PlsE species are as follows: position on the glycerol backbone as shown as sn-1/sn-2 of the fatty acid and fatty alcohol radicals (abbreviated as number of carbons: number of double bonds).

## Discussion

Fatty acids are essential constituents of cell membranes where they play multiple functions that are crucial for cell homeostasis [3], [7], [48], [49], [50]: i) as they are almost exclusively esterified to membrane phospholipids, they provide a structural support, protection, and stability to the cell, ii) together with other lipid entities such as cholesterol, they control membrane fluidity, and subsequently they influence the functioning of specific membrane proteins involved in intra- or inter-cellular signalisation as well as in the control of the flow of molecules into and out of the cell; and iii) when released by specific phospholipases, they represent powerful second messengers that are further involved in intracellular signal transduction. The essential roles played by fatty acids in cell membranes make that any quantitative or qualitative modification in tissue fatty acid composition can result in alteration of tissue and/or organ functioning. Therefore, the monitoring and the understanding of tissue fatty acid compositions appears to be essential, particularly in organs where these lipids are known to exert specific fundamental functions such as the brain, the eye, and the heart. Since access to such tissues is limited in humans, intermediate biomarkers of these lipids are needed. This is probably why the measurement of the blood lipid composition has become very common in human nutrition research as an index of the dietary fat intake but also as a biomarker of a disease risk.

Within the blood compartment, the lipid composition of plasma has often been used in human studies to consider the overall lipid status of individuals. As well described in a number of good reviews [51], [52], [53], [54], the idea behind that was that the fatty acid composition of a specific tissue would follow the same pattern than that of plasma. However, although plasma lipids might reflect the lipid status of other tissues, the fatty acid composition of plasma is also known to represent also the endogenous processing of lipids as well as their recent dietary intake [55], [56], [57], [58]. Moreover, the relationship between dietary lipids, plasma lipids and tissue concentrations in lipids was found to be non-linear and modulated by several other factors such as genetics, age, gender, and oxidative stress generated by life style (smoking, alcohol consumption, physical activity) [59], [60], [61], [62], [63], [64]. Even if they were not used routinely in a large number of studies, erythrocytes appear to be a good alternative to plasma for the measurement of circulating fatty acids since red blood cell lipids are less prone to external influence. Indeed, it is commonly said that the erythrocyte fatty acid composition is more representative than plasma for the long-term dietary intake in lipids [51], [65], [66], due to a life span of approximately 120 days [67]. Fatty acid composition of erythrocytes was used in a few number of human studies, particularly to assess the incorporation of dietary lipids into the blood compartment, and their bioavailability to peripheral tissues [4], [68], [69], [70], [71], [72], [73], [74], [75], [76], [77]. Among them, a study of Makrides and collaborators has examined the fatty compositions of the brain, the retina, and the erythrocytes from post-mortem infants [4]. By gas chromatographic analyses, the authors have shown that erythrocyte levels of DHA were positively correlated to those of the brain, the retina having not been involved in the regression analyses. Such gas chromatographic data was also obtained in rat and baboon neonates [33], [34] thus confirming ―at least for DHA― that erythrocyte fatty acid accumulation may be used as an indicator of their neural accretion. However, this relationship may be effective only over the time scale that these experiments were done, corresponding to the period of brain development. This cautionary note was clearly mentioned in two of these papers [4], [33]. Even if we have obtained our data in non-supplemented subjects, they seem to corroborate this restrictive use of red blood cell gas chromatographic profile, at least in old humans (mean age of 84.3 y). Indeed, our gas chromatography results have shown that such an association between erythrocytes and the retina, although existing, was negative (*rSpearman* = −0.733, *P* = 0.02). In addition, we have found no other relevant association between a red blood cell fatty acid and the corresponding fatty acid in the retina, except for arachidonic acid (20:4n-6) and the total n-6/n-3 ratio. The conclusions that can be drawn from these results could be, either that the associations observed during post-natal development no longer exist in the adult/elderly, or that they still do exist but they are not visible due to a global analysis processing. Indeed, gas chromatography methodology gives the relative abundance of the whole fatty acid pool in a tissue, independently from their phospholipid origin. Thus, a more precise approach would consist in LC-ESI-MS analysis that allows a quantitative evaluation of individual phospholipid entities in the different lipid fractions where the differences are likely to be more confirmed. Our results confirm that LC-ESI-MS would be a more accurate approach since they have revealed some associations between erythrocyte and ocular lipids that do not exist through gas chromatography analysis.

The results we have obtained may constitute a basis for future clinical trials related to lipids or fatty acids in ophthalmology. First, and as stated above, they may help in preferring the LC-ESI-MS technology instead of gas chromatography for lipidomic analyses. In addition, our data outline several circulating markers that can serve as a surrogate of ocular tissue lipid composition. Such markers may be useful in a wide range of retinal and/or optic nerve diseases. Among these different markers, three of them (namely PlsE18:0/20:4, PE18:0/22:4 and PE18:0/20:4+PE18:0/22:5) were positively or negatively associated with DHA in retinal ethanolamine-phospholipids. Retinal DHA is maximally concentrated in photoreceptor outer segment membrane phospholipids where it can represent up to 50% of the total esterified fatty acids [78], [79], [80], [81]. Within photoreceptor outer segments membranes, DHA is mostly found in ethanolamine- and less in choline-phospholipids [7]. As well documented by SanGiovanni and Chew [48], the functions played by DHA in retinal photoreceptors appear to be multiple and include a structural role in membranes and specific interactions with rhodopsin protein, and therefore influences visual transduction processes. In addition, DHA was shown to be the precursor of neuroprotectin D1 (NPD1), an oxygenated derivative synthesized by retinal pigment epithelium cells that shows potent anti-inflammatory and anti-apoptotic properties [82]. As reviewed by Bazan [9], a large number of evidence suggests that the integrity of photoreceptor cells is dependent at least in part to DHA provided by photoreceptors for NPD1 biosynthesis in retinal pigment epithelium cells. Such a metabolism involving DHA in retinal cells may be of great importance when considering retinal diseases exhibiting photoreceptor degeneration such as retinitis pigmentosa or AMD. The recent discovery of NPD1 and of its functions is very relevant when regarding other data showing a higher risk of the onset or progression of AMD in people consuming less DHA or DHA-rich fish [16], [17], [18], [20], [21]. These data are also very consistent with the finished or ongoing clinical trials aiming to prevent the onset or delay the progression of AMD through DHA supplementation [26], [28], [83], [84]. In such studies, one can easily imagine how interesting would be an indicator of the retinal pool of DHA, in order to link it to dietary habits (for observation studies) or to a dietary supplementation (for intervention studies), or in order to detect potent responders/non responders or compliant/non compliant subjects within the cohorts. Our results show that erythrocyte PlsE18:0/20:4, PE18:0/22:4 and PE18:0/20:4+PE18:0/22:5 would be good indicators since they were associated to 79% (for PlsE18:0/20:4) and 89% (for PE18:0/22:4 and PE18:0/20:4+PE18:0/22:5) of retinal ethanolamine-phospholipids esterified with DHA, respectively.

The use of PC16:0/20:4 as a marker of VLC-PUFA in choline-phospholipids may be more limited. Indeed, even if the presence of VLC-PUFA in human retina is now well documented [40], [85], and the relationships between VLC-PUFA and Stargardt Macular Dystrophy well established [86], [87], the exact functions of these atypical fatty acids in retinal health are still unknown. Nevertheless, PC16:0/20:4 can be used as a research tool at least to confirm the depletion of retinal VLC-PUFA in human patients or the efficacy of nutritional and non-nutritional treatments in restoring retinal VLC-PUFA [28], [88], [89]. This marker was strongly associated with retinal VLC-PUFA having the longest carbon-chain (namely PC34/6/22:6, PC36:6/22:6 and PC36:5/22:6) (*rSpearman* = −0.783, *P* = 0.01).

The data obtained on the optic nerve may be of interest regarding the pathophysiology of glaucoma. Glaucoma or glaucomatous optic neuropathy is the leading cause of blindness worldwide that will affect almost 80 million people in 2020 [13]. It is characterized by the degeneration of retinal ganglion cells whose axons form the optic nerve. The clinical diagnosis of glaucoma is based on the morphologic examination of the optic nerve head and the measurement of visual fields, meaning that retinal neuron degeneration has already started at the time of the disease discovery. This is why the identification of early markers of glaucoma represents a great challenge in order to apply early neuroprotection treatments to the optic nerve [90]. The present data reveal that several phospholipid entities in the optic nerve are associated with ethanolamine- or choline-phospholipids in red blood cells. Particularly, PlsE16:0/22:6 and PlsE16:0/22:4 in erythrocyte were negatively associated with a pool of PlsE species (namely PlsE18:0/22:5, PlsE16:0/20:3, PlsE16:0/20:4, PlsE18:0/20:4, and PlsE16:0/22:4, *rSpearman* = −0.988, *P*<0.001) representing almost 20% of total optic nerve PlsE. Previous data have shown that PlsE represent three fourth of total ethanolamine phospholipids, accounting themselves for 40% of optic nerve phospholipids [5]. The pool of optic nerve PlsE that is negatively associated to erythrocyte PlsE16:0/22:6 and PlsE16:0/22:4 would then represent about 6% of total optic nerve phospholipids. Even if its representativeness is low, one can imagine that the concentrations of these molecules would be lowered in conditions of optic nerve degeneration, making that PlsE16:0/22:6 and PlsE16:0/22:4 in erythrocytes would represent good candidates to mirror these changes. The results we have obtained on choline-phospholipids are exciting regarding one of our previous work patients with glaucoma [31]. Indeed, we have shown the occurrence of numerous changes in erythrocyte levels of several choline-phospholipid species in patients with primary open-angle glaucoma. Particularly, through linear regression analyses, we have proposed that the levels of erythrocyte concentrations of total PlsC species may start to decline about 20 years before the first clinical signs of glaucoma. Since the data of the present work show a number of positive associations between circulating and optic nerve levels of PlsC species, we can imagine a very early loss of some choline-plasmalogens in the optic nerve of patients with primary open-angle glaucoma. On the other hand, the positive associations we have found between erythrocyte levels of PC22:6/22:6 and PC20:3/22:6 with optic nerve PC25:6/22:6 and PC24:2/22:6 support the hypothesis of an additional loss of non-plasmalogen choline-phospholipids esterified with DHA in the optic nerve during glaucoma [31].

Even if this study has some originality and strength, namely the unique origin of the samples studied and on the powerful analytical methodology, it has several distinct limitations. The principal weakness is the low number of subjects included in the study. This is why the associations we have found do not point out any definitive marker of tissue composition but give a list of potential good candidates susceptible to play this role. Similar analyses should be done in a larger number of subjects including individuals known to be affected by the degenerative ophthalmic diseases mentioned in the present paper. It would be also interesting to enlarge the future analyzes to other molecules, and particularly to markers of lipid peroxidation such as Amadori–PE [91], [92], 4-hydroxynonenal, 4-hydroxyhexenal, isoprostanes, and neuroprostanes [93], [94], [95], as they could represent biomarkers of the disease state of the retina. However, and accounting to the expected demographic forecasts estimating that patients with eye diseases are expected to represent a sensitive and growing socio-economic burden in the next future, these results may be of importance when considering the increasing number of human studies related to lipids and ophthalmic diseases.

## Supporting Information

Table S1 **Complete composition in individual species of phosphatidyl-choline (PC) and plasmenyl-choline (PlsC) of erythrocytes, retinas and optic nerves issued from human donors.**
(DOC)

Table S2 **Complete composition in individual species of phosphatidyl-ethanolamine (PE) and plasmenyl- ethanolamine (PlsE) of erythrocytes, retinas and optic nerves issued from human donors.**
(DOC)
