# Supplementary material for: Lipid Composition of the Human Eye: Are Red Blood Cells a Good Mirror of Retinal and Optic Nerve Fatty Acids?
Source: PLoS One. 2012 Apr 9;7(4):e35102. doi: 10.1371/journal.pone.0035102 (PMC3322172; doi:10.1371/journal.pone.0035102)
Supplement: Table S1 — Complete composition in individual species of phosphatidyl-choline (PC) and plasmenyl-choline (PlsC) of erythrocytes, retinas and optic nerves issued from human donors. (DOC) [file pone.0035102.s001.doc]

Table S1: Concentration of individual species of phosphatidyl-choline (PC) and plasmenyl-choline (PlsC) in erythrocytes, retinas and optic nerves from human donors evaluated by liquid chromatography coupled to electrospray ionization source-mass spectrometry (LC-ESI-MS) (µg of mg phospholipids).

|  |  | **erythrocyte** | | | **retina** | | | **optic nerve** | | |
| --- | --- | --- | --- | --- | --- | --- | --- | --- | --- | --- |
|  |  | ***n=9*** | | | ***n=9*** | | | ***n=6*** | | |
|  | **[M+H]^+^** | **mean ± *SD*** | **median** | **range** | **mean ± *SD*** | **median** | **range** | **mean ± *SD*** | **median** | **range** |
| PC14:0/16:0*^a^* | 706.50 | 2.31 ± *0.88* | 2.30 | 1.36 – 3.91 | 7.16 ± *1.16* | *7.11* | 5.52 – 9.01 | 5.01 ± *0.92* | *4.91* | 4.05 – 6.39 |
| PlsC16:0/16:1 | 720.00 | 2.01 ± *0.71* | 2.11 | 1.24 – 3.48 | 2.07 ± *0.43* | *2.07* | 1.32 – 2.67 | 1.65 ± *0.57* | *1.52* | 0.87 – 2.44 |
| PC16:1/16:1 | 730.53 | *nd* | *nd* | *nd* | 1.57 ± *0.36* | *1.53* | 1.08 – 2.29 | *nd* | *nd* | *nd* |
| PC16:0/16:1 | 732.55 | 9.57 ± *4.53* | 8.63 | 5.17 – 20.80 | 17.69 ± *2.65* | *17.75* | 14.28 – 21.90 | 10.60 ± *2.30* | *10.27* | 8.26 – 14.57 |
| PC16:0/16:0 | 734.56 | 16.45 ± *5.09* | 16.55 | 6.15 – 25.48 | 60.82 ± *5.66* | *61.54* | 61.34 – 68.43 | 18.79 ± *3.56* | *17.58* | 15.58 – 23.66 |
| PlsC18:0/16:1 + PlsC16:0/18:1 + PlsC18:1/16:0 | 744.58 | 2.11 ± *0.77* | 2.43 | 1.14 – 3.07 | *nd* | *nd* | *nd* | 2.56 ± *0.53* | *2.64* | 1.78 – 3.22 |
| PlsC16:0/18:0 | 746.60 | 4.66 ± *2.01* | 4.70 | 0.84 – 8.10 | 5.00 ± *1.14* | *4.75* | 3.94 – 7.46 | 10.19 ± *2.55* | *9.90* | 6.86 – 14.16 |
| PC14:6/20:1 + PC14:5/20:2 + PC14:3/20:4 | 748.50 | *nd* | *nd* | *nd* | 2.08 ± *0.32* | *2.03* | 1.71 – 2.77 | *nd* | *nd* | *nd* |
| PC16:0/18:2 | 758.56 | 102.58 ± *17.47* | 107.89 | 79.67 – 127.33 | 13.04 ± *4.75* | *14.02* | 1.54 – 18.10 | 8.17 ± *2.03* | *7.68* | 5.96 – 14.43 |
| PC16:0/18:1 | 760.58 | 104.95 ± *17.46* | 104.63 | 84.60 – 132.64 | 158.72 ± *8.99* | *159.91* | 147.15 – 175.04 | 133.96 ± *22.09* | *132.59* | 100.38 – 167.60 |
| PC16:0/18:0 | 762.59 | 4.25 ± *0.71* | 4.11 | 3.41 – 5.61 | 24.09 ± *2.27* | *23.85* | 21.33 – 27.88 | 5.31 ± *1.65* | *4.91* | 3.67 – 8.44 |
| PlsC16:0/20:3 | 768.58 | 3.35 ± *0.62* | 3.35 | 2.55 – 4.46 | *nd* | *nd* | *nd* | *nd* | *nd* | *nd* |
| PlsC18:1/18:1 | 770.60 | *nd* | *nd* | *nd* | *nd* | *nd* | *nd* | 1.81 ± *0.41* | *1.73* | 1.32 – 2.51 |
| PlsC18:1/18:0 | 772.61 | 3.57 ± *0.60* | 3.32 | 2.74 – 4.65 | *nd* | *nd* | *nd* | 2.55 ± *0.80* | *2.39* | 1.48 – 3.88 |
| PlsC18:0/18:0 | 774.63 | 3.27 ± *0.47* | 3.24 | 2.44 – 3.99 | *nd* | *nd* | *nd* | 3.49 ± *1.03* | *3.45* | 2.00 – 5.17 |
| PC14:0/22:5 | 780.50 | 2.22 ± *1.60* | 1.57 | 1.00 – 5.88 | *nd* | *nd* | *nd* | *nd* | *nd* | *nd* |
| PC16:0/20:4 | 782.56 | 21.33 ± *4.05* | 21.53 | 14.79 – 28.12 | 11.80 ± *1.71* | *12.23* | 9.69 – 14.74 | 2.73 ± *0.53* | *2.76* | 0.06 – 3.61 |
| PC18:1/18:2 | 784.58 | 13.12 ± *2.85* | 14.34 | 7.42 – 16.10 | 7.28 ± *0.90* | *7.22* | 5.49 – 8.52 | 2.00 ± *0.64* | *1.85* | 1.27 – 2.94 |
| PC18:1/18:1 | 786.59 | 25.39 ± *5.76* | 23.67 | 19.22 – 34.49 | 11.25 ± *2.09* | *11.03* | 8.56 – 15.16 | 14.81 ± *3.51* | *14.66* | 10.30 – 20.74 |
| PC18:0/18:1 | 788.61 | 11.53 ± *1.71* | 11.25 | 8.84 – 14.31 | 35.71 ± *4.29* | *35.24* | 29.71 – 44.54 | 37.09 ± *9.89* | *37.81* | 21.14 – 51.78 |
| PlsC16:0/22:6 | 790.57 | 0.54 ± *0.14* | 0.56 | 0.36 – 0.75 | *nd* | *nd* | *nd* | *nd* | *nd* | *nd* |
| PlsC18:0/20:4 + PlsC16:0/22:4 | 794.60 | 1.79 ± *0.65* | 1.77 | 0.88 – 2.88 | *nd* | *nd* | *nd* | *nd* | *nd* | *nd* |
| PlsC18:1/20:2 | 796.61 | 1.91 ± *0.38* | 1.83 | 1.47 – 2.56 | *nd* | *nd* | *nd* | *nd* | *nd* | *nd* |
| PC18:2/20:4 + PC16:0/22:6 | 806.56 | 8.27 ± *3.60* | 8.16 | 3.42 – 15.62 | 14.31 ± *1.83* | *14.37* | 11.80 – 17.63 | *nd* | *nd* | *nd* |
| PC16:0/22:5 | 808.58 | 5.69 ± *1.41* | 5.40 | 3.86 – 8.89 | 7.63 ± *1.00* | *7.60* | 6.72 – 10.07 | 2.22 ± *0.61* | *2.15* | 1.57 – 3.37 |
| PC18:0/20:4 | 810.59 | 11.88 ± *2.46* | 12.33 | 8.43 – 14.63 | 22.57 ± *3.34* | *22.84* | 18.32 – 29.70 | 4.37 ± *1.16* | *4.01* | 3.50 – 6.59 |
| PC18:0/20:3 | 812.61 | 3.39 ± *1.02* | 3.62 | 1.90 – 4.59 | 6.02 ± *0.96* | *6.17* | 4.06 – 7.33 | 1.35 ± *0.53* | *1.26* | 0.84 – 2.22 |
| PC18:0/20:2 | 814.60 | 0.77 ± *0.37* | 0.71 | 0.48 – 1.71 | *nd* | *nd* | *nd* | 1.98 ± *0.54* | *1.86* | 1.42 – 3.01 |
| PlsC181/22:6 | 816.35 | 0.40 ± *0.12* | 0.36 | 0.30 – 0.69 | 0.45 ± *0.14* | *0.52* | 0.25 – 0.59 | 1.43 ± *0.39* | *1.37* | 1.02 – 2.04 |
| PlsC18:0/22:6 | 818.70 | 0.35 ± *0.13* | 0.34 | 0.15 – 0.54 | *nd* | *nd* | *nd* | 0.11 ± *0.07* | *0.10* | 0.03 – 0.24 |
| PlsC18:1/22:4 | 820.55 | 0.60 ± *0.15* | 0.66 | 0.37 – 0.80 | 0.65 ± *0.13* | *0.62* | 0.46 – 0.87 | 0.18 ± *0.09* | *0.15* | 0.12 – 0.35 |
| PlsC18:0/22:4 | 822.55 | 0.48 ± *0.19* | 0.48 | 0.28 – 0.90 | *nd* | *nd* | *nd* | 0.27 ± *0.11* | *0.24* | 0.16 – 0.46 |
| PlsC18:1/22:2 | 824.25 | 0.42 ± *0.12* | 0.37 | 0.29 – 0.70 | *nd* | *nd* | *nd* | 0.22 ± *0.09* | *0.23* | 0.07 – 0.34 |
| PlsC18:1/22:1 | 826.25 | 0.17 ± *0.13* | 0.13 | 0.08 – 0.47 | *nd* | *nd* | *nd* | 0.18 ± *0.05* | *0.18* | 0.10 – 0.24 |
| PC18:1/22:6 | 832.58 | 0.67 ± *0.21* | 0.63 | 0.40 – 1.03 | 3.83 ± *0.41* | *3.60* | 3.41 – 4.51 | *nd* | *nd* | *nd* |
| PC18:0/22:6 | 834.59 | 2.93 ± *1.20* | 2.67 | 1.36 – 5.67 | 33.31 ± *5.41* | *34.00* | 24.14 – 42.57 | 0.95 ± *0.32* | *0.90* | 0.61 – 1.51 |
| PC18:0/22:5 | 836.61 | 1.27 ± *0.26* | 1.20 | 0.97 – 1.76 | 6.78 ± *1.44* | *6.78* | 4.69 – 8.93 | 0.85 ± *0.31* | *0.78* | 0.56 – 1.43 |
| PC18:0/22:4 | 838.62 | 0.56 ± *0.35* | 0.39 | 0.36 – 1.44 | *nd* | *nd* | *nd* | 0.78 ± *0.26* | *0.67* | 0.55 – 1.25 |
| PC20:1/20:0 | 844.70 | *nd* | *nd* | *nd* | *nd* | *nd* | *nd* | 0.70 ± *0.0.18* | *0.72* | 0.49 – 0.93 |
| PC20:6/22:6 | 850.50 | 0.30 ± *0.22* | 0.22 | 0.09 – 0.75 | *nd* | *nd* | *nd* | *nd* | *nd* | *nd* |
| PC20:3/22:6 + PC20:4/22:5 + PC20:5/22:4 | 856.60 | 0.24 ± *0.21* | 0.15 | 0.04 – 0.71 | *nd* | *nd* | *nd* | *nd* | *nd* | *nd* |
| *unidentified* | 870.69 | *nd* | *nd* | *nd* | *nd* | *nd* | *nd* | 1.56 ± *0.55* | *1.58* | 0.73 – 2.32 |
| *unidentified* | 872.70 | *nd* | *nd* | *nd* | *nd* | *nd* | *nd* | 1.74 ± *0.55* | *1.94* | 0.97 – 2.38 |
| PC22:6/22:6 | 878.56 | 0.29 ± *0.19* | 0.27 | 0.11 – 0.75 | 1.71 ± *1.16* | *1.18* | 0.60 – 3.81 | *nd* | *nd* | *nd* |
| PC22:3/22:6 | 884.60 | *nd* | *nd* | *nd* | *nd* | *nd* | *nd* | 0.72 ± *0.31* | *0.82* | 0.28 – 1.15 |
| PC22:2/22:6 | 886.60 | *nd* | *nd* | *nd* | *nd* | *nd* | *nd* | 0.59 ± *0.16* | *0.63* | 0.30 – 0.76 |
| *unidentified* | 896.61 | *nd* | *nd* | *nd* | *nd* | *nd* | *nd* | 0.58 ± *0.29* | *0.61* | 0.18 – 1.00 |
| *unidentified* | 898.62 | *nd* | *nd* | *nd* | *nd* | *nd* | *nd* | 4.51 ± *1.73* | *4.96* | 1.88 – 6.88 |
| *unidentified* | 900.64 | *nd* | *nd* | *nd* | *nd* | *nd* | *nd* | 1.02 ± *0.38* | *1.10* | 0.41 – 1.51 |
| PC24:6/22:6 | 906.60 | *nd* | *nd* | *nd* | 0.14 ± *0.06* | *0.14* | 0.05 – 0.23 | *nd* | *nd* | *nd* |
| PC24:5/22:6 | 908.60 | *nd* | *nd* | *nd* | 0.15 ± *0.11* | *0.13* | <0.01 – 0.39 | *nd* | *nd* | *nd* |
| PC24:4/22:6 | 910.60 | *nd* | *nd* | *nd* | 0.16 ± *0.07* | *0.15* | 0.07 – 0.27 | *nd* | *nd* | *nd* |
| PC24:3/22:6 | 912.60 | *nd* | *nd* | *nd* | *nd* | *nd* | *nd* | 0.65 ± *0.34* | *0.60* | 0.20 – 1.18 |
| PC25:6/22:6 | 920.60 | *nd* | *nd* | *nd* | *nd* | *nd* | *nd* | 0.54 ± *0.39* | *0.38* | 0.22 – 1.22 |
| PC25:5/22:6 | 922.60 | *nd* | *nd* | *nd* | *nd* | *nd* | *nd* | 0.17 ± *0.09* | *0.15* | 0.05 – 0.32 |
| PC25:3/22:6 | 926.70 | *nd* | *nd* | *nd* | *nd* | *nd* | *nd* | 1.75 ± *0.57* | *1.79* | 0.89 – 2.52 |
| PC25:2/22:6 | 928.70 | *nd* | *nd* | *nd* | *nd* | *nd* | *nd* | 0.28 ± *0.22* | *0.23* | <0.01 – 0.63 |
| PC26:1/22:6 | 944.70 | *nd* | *nd* | *nd* | 0.61 ± *0.06* | *0.61* | 0.48 – 0.73 | 10.54 ± *0.11* | *0.55* | 0.34 – 0.67 |
| PC30:1/22:6 | 1000.70 | *nd* | *nd* | *nd* | 0.49 ± *0.51* | *0.35* | 0.08 – 1.78 | *nd* | *nd* | *nd* |
| PC30:0/22:6 | 1002.70 | *nd* | *nd* | *nd* | 0.34 ± *0.14* | *0.36* | 0.05 – 0.50 | *nd* | *nd* | *nd* |
| PC32:6/22:6 | 1018.80 | *nd* | *nd* | *nd* | 1.33 ± *0.66* | *1.53* | 0.51 – 2.51 | *nd* | *nd* | *nd* |
| PC32:5/22:6 | 1020.80 | *nd* | *nd* | *nd* | 1.29 ± *0.62* | *1.49* | 0.47 – 2.47 | *nd* | *nd* | *nd* |
| PC32:4/22:6 | 1022.80 | *nd* | *nd* | *nd* | 1.33 ± *0.79* | *1.17* | 0.42 – 2.86 | *nd* | *nd* | *nd* |
| PC32:3/22:6 | 1024.80 | *nd* | *nd* | *nd* | 0.83 ± *0.42* | *0.82* | 0.29 – 1.47 | *nd* | *nd* | *nd* |
| PC34:6/22:6 | 1046.80 | *nd* | *nd* | *nd* | 1.71 ± *0.80* | *1.85* | 0.06 – 3.02 | *nd* | *nd* | *nd* |
| PC34:5/22:6 | 1048.80 | *nd* | *nd* | *nd* | 3.98 ± *1.12* | *4.21* | 2.23 – 5.72 | *nd* | *nd* | *nd* |
| PC36:6/22:6 | 1074.80 | *nd* | *nd* | *nd* | 0.80 ± *0.35* | *0.78* | 0.38 – 1.58 | *nd* | *nd* | *nd* |
| PC36:5/22:6 | 1076.90 | *nd* | *nd* | *nd* | 0.50 ± *0.26* | *0.54* | 0.18 – 0.88 | *nd* | *nd* | *nd* |
| Total PC + PlsC | - | 375.66 ± *35.84* | *373.78* | 324.12 – 438.34 | 469.25 ± *21.32* | *476.78* | 424.81 – 498.77 | 290.96 ± *58.31* | *280.86* | 210.27 – 388.44 |
| Total PC + PlsC with 22:6 | - | 14.00 ± *5.02* | *13.66* | 6.85 – 24.42 | 52.99 ± *9.61* | *55.00* | 36.71 – 65.39 | 7.74 ± *2.43* | *7.62* | 4.46 – 11.64 |
| Total PlsC | - | 25.70 ± *4.34* | *25.20* | 20.75 – 35.38 | 8.18 ± *1.45* | *7.79* | 6.82 – 11.39 | 24.63 ± *6.05* | *23.56* | 16.86 – 34.88 |

*^a^*: Abbreviations of individual PC and PlsC species are as follows: position on the glycerol backbone as shown as sn-1/sn-2 of the fatty acid and fatty alcohol radicals (abbreviated as number of carbons: number of double bonds).
