# Supplementary material for: Lipid Composition of the Human Eye: Are Red Blood Cells a Good Mirror of Retinal and Optic Nerve Fatty Acids?
Source: PLoS One. 2012 Apr 9;7(4):e35102. doi: 10.1371/journal.pone.0035102 (PMC3322172; doi:10.1371/journal.pone.0035102)
Supplement: Table S2 — Complete composition in individual species of phosphatidyl-ethanolamine (PE) and plasmenyl- ethanolamine (PlsE) of erythrocytes, retinas and optic nerves issued from human donors. (DOC) [file pone.0035102.s002.doc]

Table S2: Concentration of individual species of phosphatidyl-ethanolamine (PE) and plasmenyl- ethanolamine (PlsE) in erythrocytes, retinas and optic nerves from human donors evaluated by liquid chromatography coupled to electrospray ionization source-mass spectrometry (LC-ESI-MS). Results are expressed as µg of mg phospholipids for PE species and as ratio to internal standard PC14:0/14:0 for PlsE species.

|  |  | **erythrocyte** | | | **retina** | | | **optic nerve** | | |
| --- | --- | --- | --- | --- | --- | --- | --- | --- | --- | --- |
|  |  | ***n=9*** | | | ***n=9*** | | | ***n=6*** | | |
|  | **[M+H]+ or MS/MS transition*^a^*** | **mean ± *SD*** | **median** | **range** | **mean ± *SD*** | **median** | **range** | **mean ± *SD*** | **median** | **range** |
| PE14:1/16:2 + PE14:2/:16:1*^b^* | 658.40 | *nd* | *nd* | *nd* | 0.05 ± *0.03* | *0.05* | 0.02 – 0.09 | *nd* | *nd* | *nd* |
| PE16:0/16:1 | 690.53 | 0.59 ± *0.30* | *0.69* | 0.20 – 0.96 | *nd* | *nd* | *nd* | 0.39 ± *0.32* | *0.32* | 0.09 – 0.83 |
| PE16:0/16:0 | 692.54 | 0.35 ± *0.14* | *0.35* | 0.16 – 0.55 | 0.21 ± *0.18* | *0.19* | 0.03 – 0.43 | *nd* | *nd* | *nd* |
| PE16:1/18:2 | 714.50 | 0.54 ± *0.14* | *0.57* | 0.40 – 0.74 | *nd* | *nd* | *nd* | *nd* | *nd* | *nd* |
| PE16:0/18:2 | 716.54 | 7.51 ± *1.40* | *7.04* | 6.24 – 9.92 | 2.11 ± *1.20* | *1.97* | 0.80 – 3.70 | 1.86 ± *0.91* | *1.60* | 1.11 – 3.11 |
| PE16:0/18:1 | 718.56 | 23.47 ± *4.60* | *22.65* | 19.45 – 31.35 | 8.19 ± *2.04* | *8.47* | 5.52 – 10.32 | 8.32 ± *0.76* | *8.11* | 5.26 – 11.80 |
| PE16:0/18:0 | 720.57 | 0.02 ± *0.40* | *<0.01* | <0.01 – 0.26 | *nd* | *nd* | *nd* | *nd* | *nd* | *nd* |
| PE16:1/20:4 | 738.50 | 1.34 ± *0.43* | *1.29* | 1.01 – 20.6 | *nd* | *nd* | *nd* | *nd* | *nd* | *nd* |
| PE16:0/20:4 | 740.54 | 16.65 ± *2.19* | *15.96* | 14.11 – 20.04 | 3.75 ± *1.42* | *3.80* | 2.09 – 5.28 | 1.62 ± *0.75* | *1.40* | 1.01 – 2.64 |
| PE18:1/18:2 | 742.56 | 7.42 ± *1.18* | *7.71* | 6.19 – 8.67 | *2.54 ± 0.71* | *2.74* | 1.53 – 3.17 | *2.54 ± 0.49* | *2.44* | 2.12 – 3.15 |
| PE18:1/18:1 + PE18:0/18:2 | 744.57 | 11.93 ± *1.38* | *11.89* | 10.02 – 13.93 | 9.92 ± *4.15* | *11.38* | 4.00 – 12.92 | 28.91 ± 6.94 | *30.40* | 19.70 – 35.14 |
| PE18:0/18:1 | 746.59 | 7.17 ± *1.40* | *7.92* | 4.77 – 8.14 | 14.78 ± *3.94* | *15.20* | 9.64 – 19.23 | 19.26 ± 5.75 | *19.24* | 12.24 – 26.31 |
| PE18:0/18:0 | 748.55 | <0.01 ± *0.22* | *<0.01* | <0.01 – 0.29 | *nd* | *nd* | *nd* | *nd* | *nd* | *nd* |
| PE16:0/22:6 | 764.54 | 11.69 ± *4.74* | *10.30* | 6.47 – 19.34 | 31.15 ± *13.39* | *26.90* | 20.22 – 50.68 | 2.07 ± *0.98* | *1.80* | 1.20 – 3.46 |
| PE18:1/20:4 + PE16:0/22:5 | 766.56 | 16.61 ± *2.64* | *15.09* | 14.10 – 20.24 | 11.73 ± *2.14* | *11.31* | 9.60 – 14.70 | 8.71 ± *2.53* | *8.14* | 6.67 – 11.92 |
| PE18:0/20:4 | 768.57 | 21.53 ± *2.53* | *20.59* | 19.30 – 24.47 | 54.35 ± *6.38* | *53.53* | 47.52 – 62.83 | 17.78 ± *2.85* | *18.11* | 14.15 – 20.76 |
| PE18:0/20:3 | 770.59 | 0.33 ± *0.36* | *0.38* | <0.01 – 0.88 | 7.00 ± *2.45* | *7.17* | 3.85 – 9.80 | 4.24 ± *1.84* | *4.67* | 1.81 – 5.78 |
| PE18:0/20:2 + PE18:1/20:1 | 772.60 | 0.42 ± *0.06* | *0.40* | 0.38 – 0.52 | *nd* | *nd* | *nd* | 8.86 ± *2.82* | *9.19* | 5.25 – 11.80 |
| PE18:0/20:1 | 774.56 | 0.35 ± *0.13* | *0.40* | 0.16 – 0.46 | *nd* | *nd* | *nd* | 3.96 ± *1.17* | *3.77* | 2.76 – 5.53 |
| PE18:0/20:0 | 776.50 | *nd* | *nd* | *nd* | 6.97 ± *2.95* | *5.87* | 4.85 – 11.29 | *nd* | *nd* | *nd* |
| PE20:4/20:4 | 788.54 | 1.36 ± *0.58* | *1.39* | 0.68 – 1.93 | *nd* | *nd* | *nd* | *nd* | *nd* | *nd* |
| PE18:1/22:6 | 790.56 | 3.89 ± *1.62* | *3.43* | 1.90 – 6.31 | 26.37 ± *18.20* | *19.79* | 12.83 – 53.08 | *2.07 ± 0.51* | *1.97* | 1.57 – 2.76 |
| PE18:0/22:6 | 792.57 | 4.52 ± *2.07* | *3.49* | 2.36 – 7.47 | 177.78 ± *79.67* | *149.50* | 116.65 – 294.27 | 9.82 ± *0.76* | *9.85* | 8.92 – 10.66 |
| PE18:0/22:5 | 794.59 | 3.79 ± *0.93* | *3.53* | 3.01 – 5.21 | *nd* | *nd* | *nd* | 7.83 ± *2.23* | *7.97* | 4.97 – 10.40 |
| PE18:0/22:4 | 796.60 | 2.42 ± *0.64* | *2.47* | 2.19 – 3.42 | 12.03 ± *3.57* | *13.61* | 6.69 – 14.21 | 6.43 ± *1.66* | *4.72* | 4.34 – 7.96 |
| PE20:0/20:3 + PE22:1/18:2 | 798.62 | 0.07 ± *0.05* | *0.08* | <0.01 – 0.13 | *nd* | *nd* | *nd* | 2.52 ± *0.54* | *2.43* | 1.97 – 3.25 |
| PE20:2/20:0 | 800.65 | *nd* | *nd* | *nd* | *nd* | *nd* | *nd* | 1.82 ± *1.08* | *1.56* | 0.90 – 3.25 |
| *unidentified* | 806.60 | *nd* | *nd* | *nd* | 0.70 ± *0.58* | *0.65* | 0.09 – 1.41 | *nd* | *nd* | *nd* |
| PE20:4/22:6 | 812.52 | 0.44 ± *0.28* | *0.33* | 0.16 – 0.89 | 2.40 ± *0.88* | *2.56* | 1.32 – 3.17 | *nd* | *nd* | *nd* |
| PE20:3/22:6 | 814.56 | 0.24 ± *0.15* | *0.27* | 0.01 – 0.43 | 5.20 ± *4.74* | *3.98* | 1.33 – 11.50 | *nd* | *nd* | *nd* |
| PE20:2/22:6 + PE20:4/22:4 | 816.50 | *nd* | *nd* | *nd* | 2.31 ±*1.26* | *2.27* | 0.82 – 3.87 | *nd* | *nd* | *nd* |
| PE20:1/22:6 | 818.56 | 0.23 ± *0.36* | *0.06* | <0.01 – 0.84 | 7.05 ± *5.63* | *4.62* | 3.50 – 15.44 | *nd* | *nd* | *nd* |
| *PE20:0/22:6 + PE20:2/22:4* | 820.55 | *nd* | *nd* | *nd* | 1.57 ± *0.72* | *1.51* | 0.77 – 2.50 | *nd* | *nd* | *nd* |
| *PE20:0/22:4* | 824.63 | *nd* | *nd* | *nd* | 0.49 ± *0.27* | *0.52* | 0.14 – 0.79 | 1.69 ± *0.91* | *1.60* | 0.78 – 2.81 |
| PE22:6/22:6 | 836.56 | *nd* | *nd* | *nd* | 2.81 ± *1.38* | *2.91* | 1.10 – 4.34 | *nd* | *nd* | *nd* |
| PE22:5/22:6 | 838.60 | *nd* | *nd* | *nd* | 2.32 ± *0.48* | *2.30* | 1.77 – 2.89 | *nd* | *nd* | *nd* |
| PE22:4/22:6 | 840.50 | *nd* | *nd* | *nd* | 4.22 ± *2.01* | *4.03* | 2.04 – 6.79 | *nd* | *nd* | *nd* |
| PE22:3/22:6 | 842.60 | *nd* | *nd* | *nd* | 1.52 ± *0.85* | *1.58* | 0.56 – 2.35 | 0.22 ± *0.11* | *0.24* | 0.07 – 0.32 |
| PE22:2/22:6 | 844.60 | *nd* | *nd* | *nd* | *nd* | *nd* | *nd* | 0.28 ± *0.07* | *0.29* | 0.18 – 0.34 |
| PlsE16:0/20:4 | 722 ->303 | 0.25 ± *0.14* | *0.27* | <0.01 – 0.36 | 0.21 ± *0.03* | *0.21* | 0.17 – 0.25 | 0.16 ± *0.10* | *0.11* | 0.10 – 0.31 |
| PlsE16:0/20:3 | 724 -> 305 | 0.02 ± *0.01* | *0.03* | <0.01 – 0.03 | 0.03 ± *0.01* | *0.03* | 0.02 – 0.03 | 0.07 ± *0.02* | *0.07* | 0.05 – 0.09 |
| PlsE18:0/18:1 | 728 -> 281 | 0.27 ± *0.16* | *0.28* | 0.05 – 0.43 | 0.21 ± *0.04* | *0.22* | 0.16 – 0.25 | 1.15 ± *0.24* | *1.09* | 0.93 – 1.36 |
| PlsE16:0/22:6 | 746 -> 327 | 0.02 ± *0.01* | *0.01* | <0.01 – 0.02 | 0.05 ± *0.01* | *0.05* | 0.04 – 0.06 | 0.01 ± *<0.01* | *0.01* | <0.01 – 0.02 |
| PlsE18:1/20:4 | 748 -> 303 | 0.20 ± *0.12* | *0.25* | 0.01 – 0.29 | 0.17 ± *0.02* | *0.17* | 0.13 – 0.19 | 0.46 ± *0.10* | *0.49* | 0.32 – 0.54 |
| PlsE16:0/22:5 | 748 -> 329 | 0.11 ± *0.06* | *0.13* | <0.01 – 0.15 | 0.03 ± *0.01* | *0.03* | 0.03 – 0.04 | 0.04 ± *0.01* | *0.04* | 0.03 – 0.05 |
| PlsE18:0/20:4 | 750 -> 303 | 0.71 ± *0.39* | *0.81* | 0.05 – 0.98 | 0.71 ± *0.09* | *0.70* | 0.62 – 0.82 | 0.27 ± *0.05* | *0.25* | 0.24 – 0.34 |
| PlsE16:0/22:4 | 750 -> 331 | 0.28 ± *0.16* | *0.35* | <0.01 – 0.36 | 0.08 ± *0.01* | *0.08* | 0.06 – 0.09 | 0.54 ± *0.10* | *0.58* | 0.39 – 0.59 |
| PlsE18:1/20:2 | 752 -> 307 | *nd* | *nd* | *nd* | *nd* | *nd* | *nd* | 0.21 ± *0.05* | *0.24* | 0.16 – 0.25 |
| PlsE18:1/20:1 | 754 -> 309 | *nd* | *nd* | *nd* | *nd* | *nd* | *nd* | 1.36 ± *0.37* | *1.41* | 0.92 – 1.66 |
| PlsE18:0/20:2 | 754 -> 307 | *nd* | *nd* | *nd* | <0.01 ± *<0.01* | *<0.01* | <0.01 - <0.01 | 0.09 ± *0.03* | *0.10* | 0.06 – 0.11 |
| PlsE18:1/22:6 | 772 -> 327 | 0.01 ± *<0.1* | *0.02* | <0.01 – 0.02 | 0.02 ± *<0.01* | *0.02* | 0.01 – 0.02 | <0.01 ± *<0.01* | *<0.01* | <0.01 – 0.01 |
| PlsE18:0/22:6 | 774 -> 327 | 0.04 ± *0.03* | *0.06* | <0.01 – 0.06 | 0.18 ± *0.03* | *0.17* | 0.14 – 0.21 | 0.05 ± *<0.01* | *0.05* | 0.04 – 0.05 |
| PlsE18:1/22:4 | 776 ->331 | 0.08 ± *0.05* | *0.09* | <0.01 – 0.12 | 0.02 ± *<0.01* | *0.02* | 0.02 – 0.02 | 0.97 ± *0.18* | *1.03* | 0.74 – 1.11 |
| PlsE18:0/22:5 | 776 ->329 | 0.18 ± *0.11* | *0.20* | 0.01 – 0.27 | 0.11 ± *0.02* | *0.10* | 0.09 – 0.13 | 0.06 *± <0.01* | *0.06* | 0.05 – 0.06 |
| PlsE18:0/22:4 | 778 ->329 | 0.04 ± *<0.01* | *0.04* | 0.03 – 0.05 | 0.03 ± *<0.01* | *0.03* | 0.03 – 0.04 | <0.01 ± *<0.01* | *<0.01* | 0.01 – 0.01 |
| PlsE18:1/20:2 | 780 ->335 | *nd* | *nd* | *nd* | *nd* | *nd* | *nd* | 0.16 ± *0.04* | *0.19* | 0.13 – 0.19 |
| Total PE | - | 144.85 *± 23.99* | *129.72* | 124.33 – 185.96 | 400.72 ± 141.60 | *350.84* | 292.42 – 608.75 | 141.17 ± *30.26* | *148.74* | 99.01 – 168.80 |
| Total PE with 22:6 |  | 21.00 *± 8.88* | *17.83* | 10.95 – 34.68 | 260.51 ± 124.40 | *218.27* | 163.16 – 442.35 | 12.38 ± *0.80* | *12.46* | 11.36 – 13.26 |
| Total PlsE | - | 3.95 *± 2.55* | *4.56* | 0.33 – 6.24 | 1.85 ± 0.24 | *1.83* | 1.59 – 2.15 | 5.60 ± *1.02* | *5.92* | 4.21 – 6.37 |
| Total PlsE with 22:6 | - | 0.07 *± 0.04* | *0.10* | <0.01 – 0.10 | 0.25 ± 0.04 | *0.25* | 0.20 – 0.30 | 0.06 ± *<0.01* | *0.06* | 0.06 – 0.07 |

*^a^*: [M+H]+ for PE species and MS/MS transition for PlsE species

*^b^*: Abbreviations of individual PE and PlsE species are as follows: position on the glycerol backbone as shown as sn-1/sn-2 of the fatty acid and fatty alcohol radicals (abbreviated as number of carbons: number of double bonds).
